# The karrikin receptor KAI2 promotes drought resistance in *Arabidopsis thaliana*

**DOI:** 10.1371/journal.pgen.1007076

**Published:** 2017-11-13

**Authors:** Weiqiang Li, Kien Huu Nguyen, Ha Duc Chu, Chien Van Ha, Yasuko Watanabe, Yuriko Osakabe, Marco Antonio Leyva-González, Mayuko Sato, Kiminori Toyooka, Laura Voges, Maho Tanaka, Mohammad Golam Mostofa, Motoaki Seki, Mitsunori Seo, Shinjiro Yamaguchi, David C. Nelson, Chunjie Tian, Luis Herrera-Estrella, Lam-Son Phan Tran

**Affiliations:** 1 Signaling Pathway Research Unit, RIKEN Center for Sustainable Resource Science, Yokohama, Japan; 2 Faculty of Bioscience and Bioindustry, Tokushima University, Tokushima, Japan; 3 Deutsche Forschungsgemeinschaft Center for Regenerative Therapies, Technische Universität Dresden, Fetscherstraße 105, Germany; 4 Mass Spectrometry and Microscopy Unit, RIKEN Center for Sustainable Resource Science, Yokohama, Japan; 5 Department of Genetics, University of Georgia, Athens, Georgia, United States of America; 6 Plant Genomic Network Research Team, RIKEN Center for Sustainable Resource Science, Yokohama, Japan; 7 Dormancy and Adaptation Research Unit, RIKEN Center for Sustainable Resource Science, Yokohama, Japan; 8 Department of Biomolecular Sciences, Graduate School of Life Sciences, Tohoku University, Sendai, Miyagi, Japan; 9 Department of Botany & Plant Sciences, University of California, Riverside, Riverside, California, United States of America; 10 Key Laboratory of Mollisols Agroecology, Northeast Institute of Geography and Agroecology, Chinese Academy of Sciences, Changchun, People's Republic of China; 11 Laboratorio Nacional de Genómica para la Biodiversidad (Langebio)/Unidad de Genómica Avanzada, Centro de Investigación y Estudios Avanzados del Instituto Politécnico Nacional, Irapuato, Guanajuato, Mexico; University of California, San Diego, UNITED STATES

## Abstract

Drought causes substantial reductions in crop yields worldwide. Therefore, we set out to identify new chemical and genetic factors that regulate drought resistance in *Arabidopsis thaliana*. Karrikins (KARs) are a class of butenolide compounds found in smoke that promote seed germination, and have been reported to improve seedling vigor under stressful growth conditions. Here, we discovered that mutations in *KARRIKIN INSENSITIVE2* (*KAI2*), encoding the proposed karrikin receptor, result in hypersensitivity to water deprivation. We performed transcriptomic, physiological and biochemical analyses of *kai2* plants to understand the basis for *KAI2*-regulated drought resistance. We found that *kai2* mutants have increased rates of water loss and drought-induced cell membrane damage, enlarged stomatal apertures, and higher cuticular permeability. In addition, *kai2* plants have reduced anthocyanin biosynthesis during drought, and are hyposensitive to abscisic acid (ABA) in stomatal closure and cotyledon opening assays. We identified genes that are likely associated with the observed physiological and biochemical changes through a genome-wide transcriptome analysis of *kai2* under both well-watered and dehydration conditions. These data provide evidence for crosstalk between ABA- and KAI2-dependent signaling pathways in regulating plant responses to drought. A comparison of the strigolactone receptor mutant *d14* (*DWARF14*) to *kai2* indicated that strigolactones also contributes to plant drought adaptation, although not by affecting cuticle development. Our findings suggest that chemical or genetic manipulation of *KAI2* and *D14* signaling may provide novel ways to improve drought resistance.

## Author summary

MORE AXILLARY GROWTH2 (MAX2) is a central regulator of both strigolactone and karrikin (KAR) signaling pathways, which control many aspects of plant growth and development. MAX2 promotes plant resistance to drought. Application of a strigolactone analog enhances plant adaptation to drought, implying that strigolactone signaling regulates this trait; however, the potential contributions of the KAR signaling pathway to plant drought responses have not been explored. In this study, the functions of the KAR receptor KARRIKIN INSENSITIVE2 (KAI2) were analyzed to elucidate the role of KAI2-mediated signaling in plant drought adaptation. Our results indicate that the KAI2 signaling positively regulates drought resistance of *Arabidopsis* plants by both drought avoidance and drought tolerance strategies, such as through enhancing cuticle formation, stomatal closure, cell membrane integrity and anthocyanin biosynthesis. Our findings provide potential for manipulation of KAR- and/or strigolactone-mediated signaling pathways for improvement of drought resistance.

## Introduction

Water deficit is a major constraint to crop productivity worldwide. As water resources are increasingly challenged by climate change and the demands of a growing human population, improvement of water use efficiency and development of drought-resistant crops will be critical innovations for agriculture [1]. Intensive efforts have been made toward discovering the hormones and genetic networks that control drought adaptation in plants, with the goal of developing chemical or genetic tools to manipulate drought resistance in the field. The most well-known drought-related phytohormone is abscisic acid (ABA), which accumulates in response to drought and other abiotic stresses. ABA triggers various physical and physiological mechanisms for plant protection, including stomatal closure, cuticle formation, and production of protective metabolites [2–4]. Another phytohormone, strigolactone (SL), was recently reported to promote drought resistance in several plant species, including *Arabidopsis thaliana*, *Lotus japonicus*, and *Solanum lycopersicum*, through both ABA-dependent and ABA-independent pathways [5–8]. SLs are perceived by the α/β-hydrolase protein DWARF14 (D14), which undergoes a conformational change after SL hydrolysis that promotes interactions with the F-box protein MORE AXILLARY GROWTH2 (MAX2)/DWARF3 (D3) and downstream effectors in the SUPPRESSOR OF MAX2 1 (SMAX1)-LIKE (SMXL)/DWARF53 (D53) family [9,10]. MAX2 functions as an adapter component of an Skp1–Cullin–F-box (SCF) E3 ubiquitin ligase complex that targets D53 in rice and its orthologs in *Arabidopsis*, SMXL6/7/8, for polyubiquitination and proteasomal degradation.

Intriguingly, MAX2 is not only a central regulator of SL signaling, but is also necessary for responses to karrikins (KARs) [9,11,12]. KARs are a class of butenolide compounds found in smoke that are thought to be ecologically important triggers of seed germination in the post-fire environment [13,14]. KAR treatment has additional effects on *Arabidopsis* growth that include inhibition of hypocotyl elongation, and promotion of cotyledon expansion and greening in seedlings [13]. The KAR signaling mechanism is likely to be very similar to that of SLs. In *Arabidopsis*, KAR perception requires KARRIKIN INSENSITIVE2 (KAI2)/HYPOSENSITIVE TO LIGHT (HTL), an ancient paralog of D14 [11]. Direct association of MAX2 and KAI2 has not been demonstrated, but residues that are required for D14 interaction with MAX2 are conserved in KAI2 proteins. Epistasis tests demonstrated that SMAX1 and SMXL2, which are in the same gene family as the SL pathway targets, act downstream of MAX2 as repressors of KAR responses; therefore, they are putative substrates of a KAR-activated KAI2-SCF^MAX2^ complex [15]. It must be noted that although *Arabidopsis* KAI2 can bind KARs *in vitro* and mediate growth responses to KAR treatments, KAR perception may not be its typical function. Instead, KARs may function as natural analogs of a hypothetical, endogenous KAI2 ligand (KL). Supporting this idea, KAI2 proteins with specialized ligand preferences have been identified in root parasitic plants; a KAR-responsive *KAI2i* paralog from *Striga hermonthica* only partially rescues *Arabidopsis kai2* mutants, whereas a highly conserved *KAI2c* paralog from *Pheliphanche aegyptiaca* fully rescues *kai2* but is not responsive to KAR_1_ [16,17]. Therefore, KARs may be useful as lead compounds to develop novel agonists of KAI2 signaling that improve crop performance.

Because MAX2 functions in two pathways, *max2* phenotypes cannot be readily attributed to defects in the SL, KAR/KL, or both signaling mechanisms. Further complicating the matter, a commonly used SL analog known as GR24 is typically synthesized as a racemic mixture; one GR24 enantiomer has the stereochemical configuration of natural SLs and selectively activates D14, whereas the other has an unnatural configuration and can activate both KAI2 and D14 in *Arabidopsis* [18]. Two studies have shown that *Arabidopsis max2* mutants have less drought-resistance than wild-type (WT), but it has been unclear whether this is due to a SL signaling defect because contradictory drought-sensitive phenotypes have been reported for SL-deficient mutants [5,6]. Spraying plants with *rac*-GR24 promotes survival after water deficit in both WT and SL-deficient *max* mutants [5], but this does not exclude the possibility that this is due, at least in part, to KAI2 activation. As KAR-containing smoke-water has been shown to increase germination, seedling vigor, and survival in crops grown under stressful environments, including high temperature and low osmotic potential conditions [14,19], we hypothesized that KAI2 signaling may contribute to MAX2-dependent drought resistance. Here we demonstrate a role for *KAI2* in survival of *Arabidopsis* plants after water deficit, and identify physiological and molecular explanations for this function, suggesting the importance of an as-yet-unknown signal KL and KAI2-dependent signaling in plant drought resistance. Additionally, by analyzing the drought resistance of SL-specific *d14* and *kai2* receptor mutants we find that both pathways promote plant adaptation to water deficit.

## Results

### *kai2* mutant plants are hypersensitive to drought

**Fig 1:**
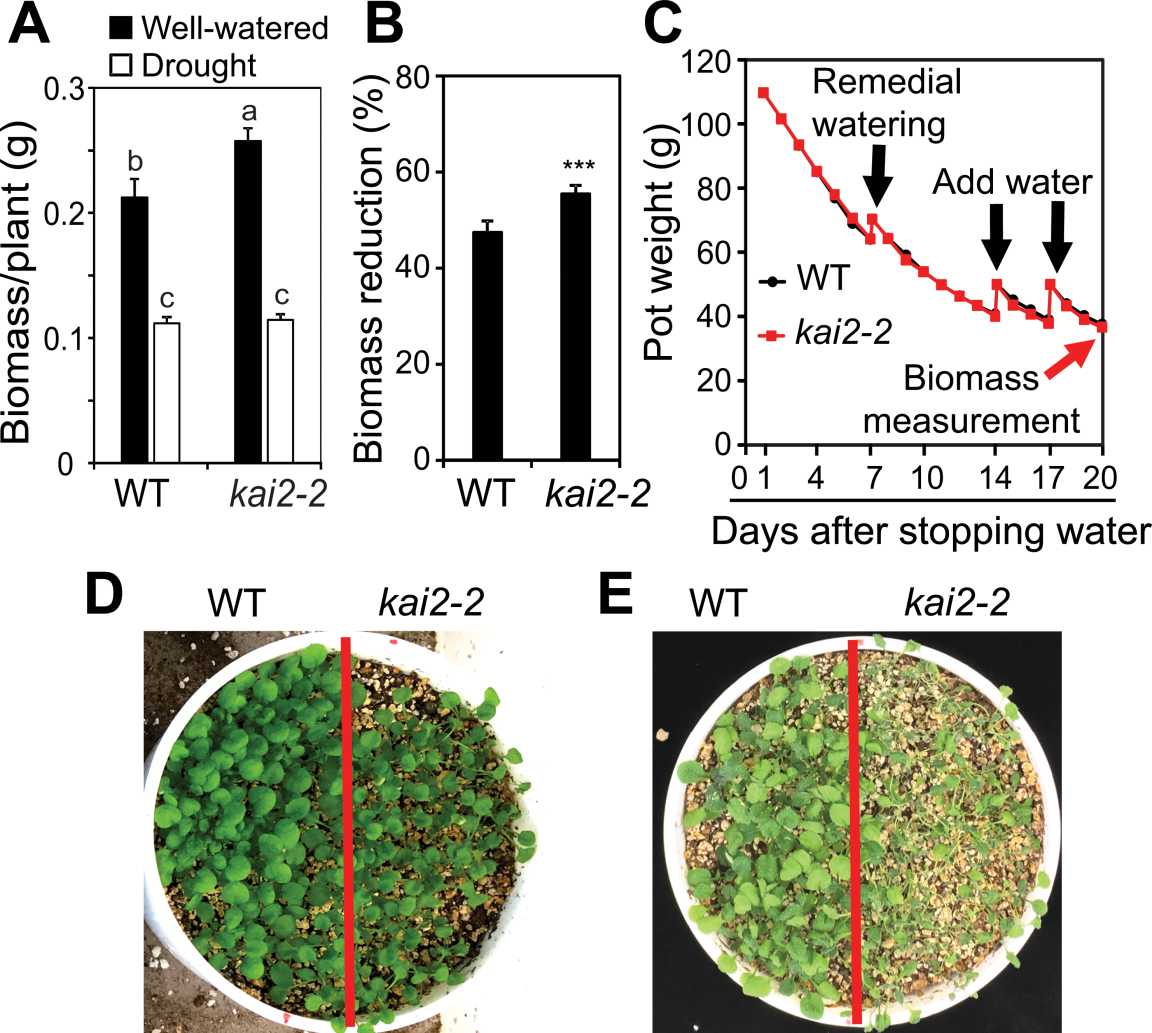
Effects of *KAI2* on drought resistance. (**A**) Biomass of *kai2-2* and WT plants under drought stress and well-watered control (*n* = 9 biological replicates). The different letters above the error bars indicate significant differences (*P* < 0.05) in all combinations according to a Tukey's honest significant difference test. (**B**) Biomass reduction of *kai2-2* and WT plants under drought stress relative to respective well-watered control. Data represent the means and standard errors (*n* = 9 biological replicates). Asterisks indicate significant differences as determined by a Student’s *t*-test, ***P < 0.001. (**C**) Averaged pot weights of *kai2-2* and WT plants during drought stress (*n* = 9 biological replicates). Black arrows indicate when water was added to the root growth area in the soil. Red arrow indicates when biomass was measured. (**D-E**) WT and *kai2-2* mutant plants were grown on water-saturated soil for 8 days. Watering was then stopped for 7 days (**D**) and 14 days (**E**).

To investigate whether KAI2-mediated signaling contributes to drought resistance in *Arabidopsis*, we examined the effects of *KAI2* loss-of-function on plant growth during water deficit using the gravimetric method [20]. We found that the biomass of *kai2-2* mutant plants was higher than that of WT when they were grown under well-watered conditions (Fig 1A). However, the biomass reduction of *kai2-2* mutants was higher than that of WT when they were grown under similar low soil water content after water withholding (Fig 1B and 1C). Additionally, when the *kai2-2* and WT plants were grown under high density in the same pots, we observed that *kai2-2* mutant plants displayed higher sensitivity to drought than WT (Fig 1D and 1E). These results together suggest that KAI2 signaling promotes adaptation to drought in *Arabidopsis*.

### Water loss, drought-induced cell membrane damage, and stomatal opening are increased in *kai2* plants

**Fig 2:**
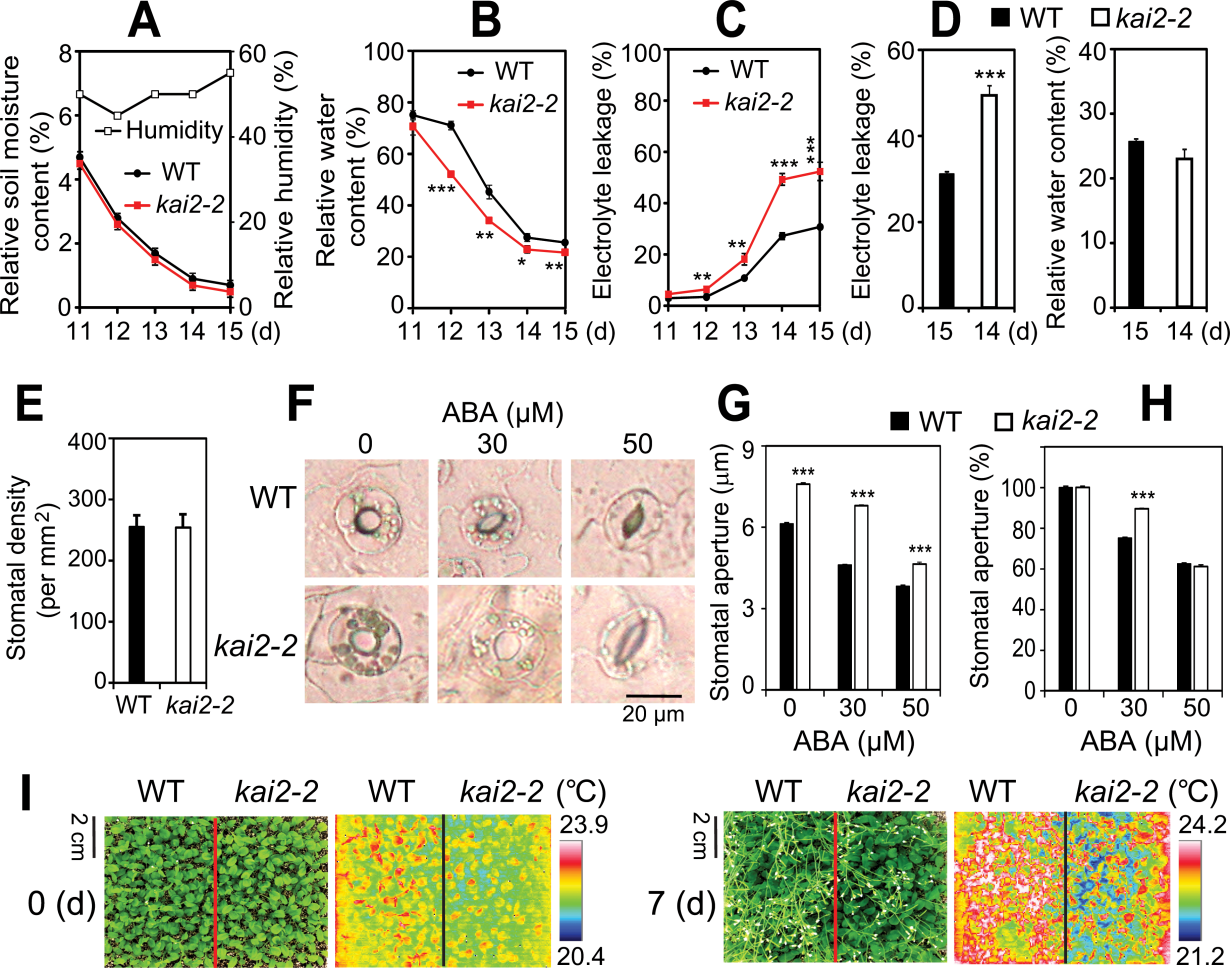
Drought-associated traits of *kai2-2* leaves. (**A-C**) *kai2-2* and WT plants were grown and exposed to drought. At indicated time points, (**A**) soil relative moisture contents (*n* = 10) and relative humidity, (**B**) leaf relative water content (RWC) (*n* = 4 biological replicates), and (**C**) electrolyte leakage (*n* = 4 biological replicates) were determined. (**D**) Electrolyte leakage (*Left*) of *kai2-2* and WT plants at a similar RWC (*Right*) during drought treatment (*n* = 4 biological replicates). Data represent the means and standard errors. (**E**) Average stomatal density of rosette leaves (abaxial side) from 14-day-old plate-grown *kai2-2* and WT plants. Error bars represent standard errors (*n* = 15 leaves/15 independent plants/genotype). (**F**) Representative guard cells of rosette leaves from 21-day-old plate-grown *kai2-2* and WT plants treated with 0, 30 and 50 μM ABA. (**G-H**) Average width of stomatal aperture of rosette leaves from 21-day-old WT and *kai2-2* plants in the presence or absence of ABA. Aperture width are shown in micrometers (**G**) or in percentage of the average aperture width obtained from absence of ABA treatment (**H**). Error bars represent standard errors (*n* = 5 plants; for each plant the average of nine stomatal measurements from a single leaf was calculated). (**I**) Leaf surface temperatures of well-watered WT and *kai2-2* plants before (0 d) and after a 7-d drought period (7 d). Plants were 21-d-old at the start of water withholding. Common optical camera (*Left*) and thermal imaging camera (*Right*) were used to take pictures at the same time. Asterisks indicate significant differences between the genotypes under well-watered control or drought conditions as determined by a Student’s *t*-test, *P < 0.05; **P < 0.01; ***P < 0.001.

We next investigated the physiological basis for drought sensitivity in *kai2-2* mutant plants. Consistent with their reduced drought resistance, both *kai2-2* and *kai2-4* plants had lower relative water content (RWC) than WT on drying soil at similar soil moisture contents (Fig 2A and 2B, S1A and S1B Fig). A faster rate of water loss during drought might be attributable to several factors, such as increases in cell permeability and/or gas exchange through stomata. We used an electrolyte leakage assay to assess cell membrane integrity, and found that both *kai2-2* and *kai2-4* plants had higher electrolyte leakage than WT during water deficit (Fig 2C and S1C Fig). Notably, *kai2-2* and *kai2-4* plants had higher electrolyte leakage than WT even at time points when both genotypes had similar RWC (Fig 2D and S1D Fig). This finding implies that the degree of cell-membrane injury induced by water deprivation is more severe in *kai2*, contributing to its drought-susceptible phenotype.

We assessed whether differences in stomatal density or movement might also affect the rate of water loss in *kai2-2* plants. Stomatal density was comparable between WT and *kai2-2* rosette leaves (Fig 2E). However, *kai2-2* had larger stomatal apertures than WT plants (Fig 2F and 2G). As ABA is induced during drought and promotes stomatal closure [21], we examined the responses of *kai2-2* and WT stomata to ABA treatment. The stomatal apertures of *kai2-2* plants were significantly larger than WT in the presence of 30 and 50 μM ABA (Fig 2F and 2G). Normalizing the ABA-treated data against the untreated apertures of WT and *kai2-2* stomata indicated that *kai2-2* stomata undergo a similar degree of closure as WT in the presence of 50 μM ABA, but are somewhat less sensitive to an intermediate (30 μM) ABA concentration (Fig 2H).

These results together suggested that *kai2* plants may have increased transpiration rates during water deficit. We used leaf surface temperature measurements as a proxy to compare transpiration between *kai2* and WT plants. We found that the leaf surfaces of both *kai2-2* and *kai2-4* plants were remarkably cooler than WT before and after 7 d of water withholding (Fig 2I and S1E Fig). Our findings collectively indicate that the differences in RWC due to stomatal water losses enhance the susceptibility of *kai2* plants to drought.

### *KAI2* promotes ABA responses and ABA catabolism

**Fig 3:**
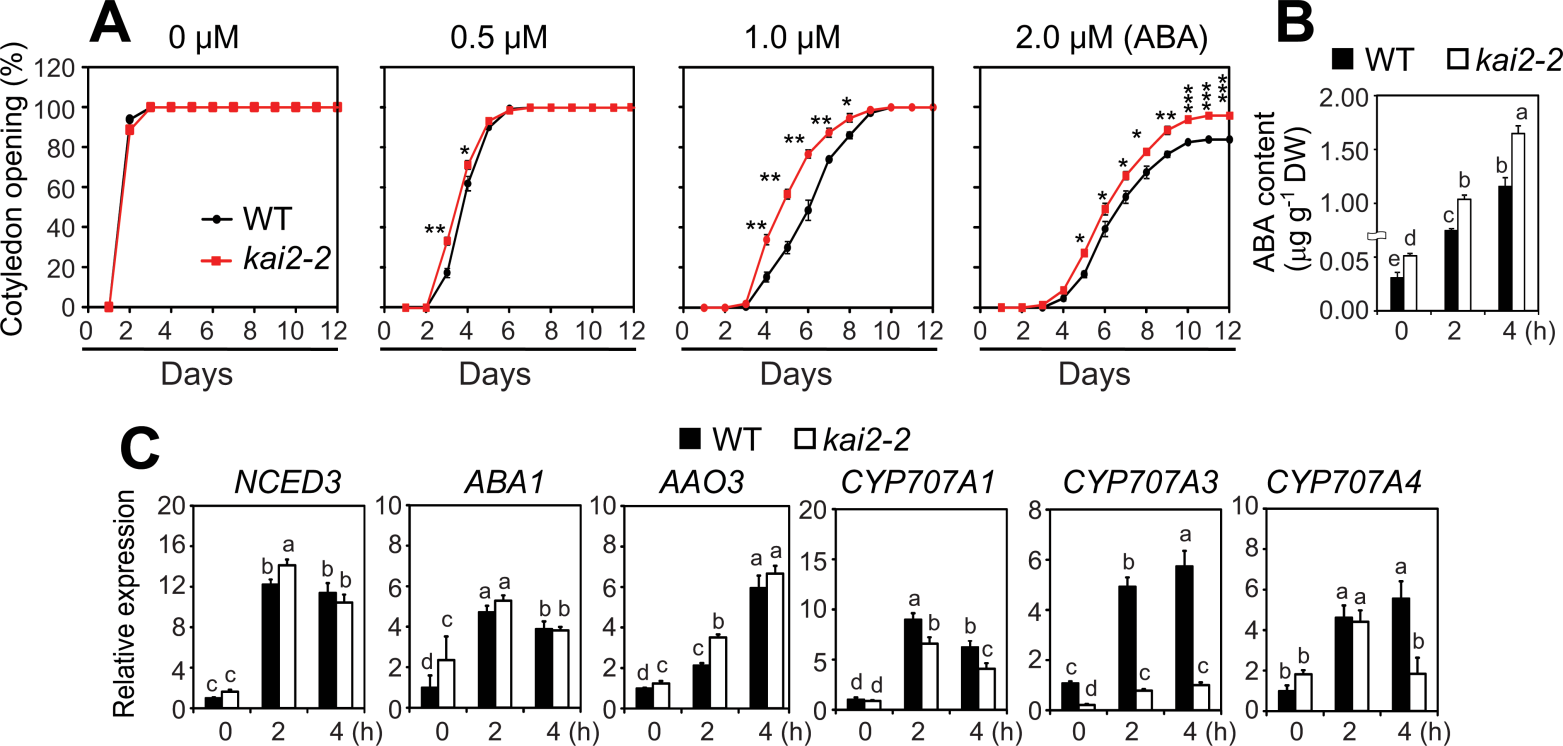
KAI2 effects on ABA responses and metabolism. (**A**) Cotyledon opening percentage of *kai2-2* and WT seedlings in the absence or presence of different concentrations of exogenous ABA. Data represent the means and standard deviation of 3 independent experiments (*n* = 50 seeds/genotype/experiment). Asterisks indicate significant differences as determined by a Student’s *t*-test, *P < 0.05; **P < 0.01; ***P < 0.001. (**B**) Endogenous ABA contents in leaves of 24-d-old *kai2-2* and WT plants under normal and dehydration conditions. Data represent the means and standard errors (*n* = 5 plants). (**C**) Expression of genes involved in ABA biosynthesis and catabolism in leaves of 24-day-old *kai2-2* and WT plants under normal and dehydration conditions. Relative expression levels were normalized to a value of 1 in the WT grown under normal conditions. Data represent the means and standard errors (*n* = 5 biological replicates). The different letters above the error bars indicate significant differences (*P* < 0.05) in all combinations according to a Tukey's honest significant difference test.

The partial reduction in ABA-induced stomatal closure in the *kai2* mutant raised the question of whether ABA responses are broadly impaired, which would likely impact drought adaptation [21]. To test this, we compared the inhibitory effects of ABA on cotyledon opening of *kai2* and WT seedlings. While the cotyledon opening rates of *kai2-2* and *kai2-4* mutants were similar to WT in the absence of ABA, both *kai2* alleles showed decreased sensitivity to ABA treatments (Fig 3A and S1F Fig). Thus, KAI2 positively regulates multiple ABA responses in *Arabidopsis*.

Next, we investigated whether *KAI2* might also influence endogenous ABA levels in plants, especially under drought. We measured ABA content in the leaves of *kai2-2* and WT plants during a 4 h time course of dehydration. Interestingly, *kai2-2* leaves had significantly increased endogenous ABA contents than WT before and during dehydration (Fig 3B). To determine whether this was due to differential regulation of ABA metabolism-related genes, we analyzed the expression of several genes involved in ABA biosynthesis (*NCED3*, *ABA1*, and *AAO3*) and catabolism (*CYP701A1*, *3*, and *4*) with quantitative real-time RT-PCR (qRT-PCR). The most striking finding was that *CYP707A3* transcripts were reduced several-fold in both well-watered and dehydrated *kai2-2* plants in comparison to WT controls (Fig 3C). *CYP707A3* encodes a major 8'-hydroxylase of ABA that is highly induced during dehydration [22]. Loss of *CYP707A3* increases ABA content but, in contrast to *kai2-2*, causes ABA hypersensitivity [22]. Putatively, decreased sensitivity to endogenous ABA in *kai2-2* might shift ABA homeostasis to higher levels through feedback reduction of ABA catabolism.

### Comparative transcriptome analysis of KAI2 function during dehydration

**Fig 4:**
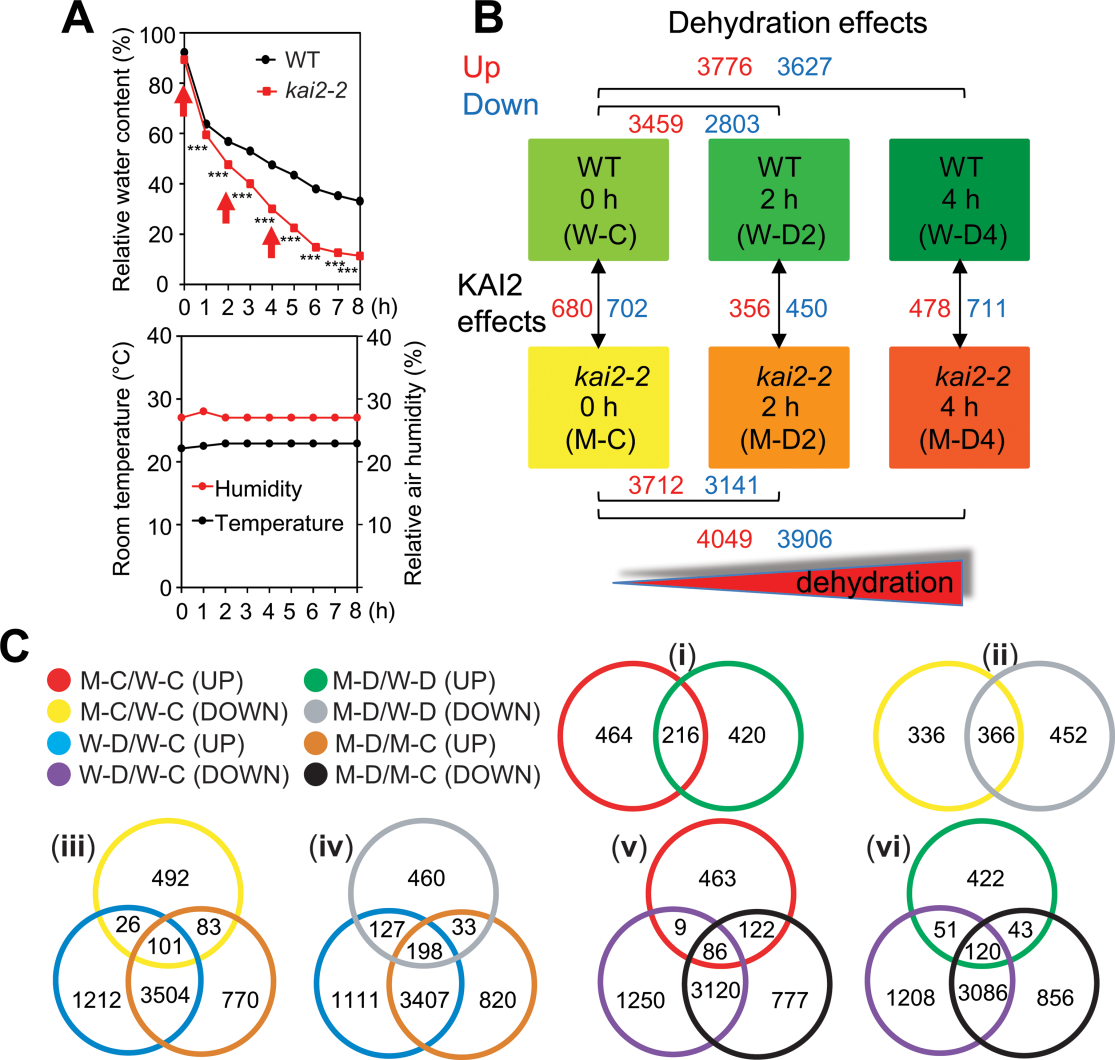
Transcriptome analysis of *kai2-2* and WT plants under normal and dehydration conditions. (**A**) Relative water content (RWC) of leaves from 24-d-old well-watered *kai2-2* and WT plants exposed to dehydration treatment. Data represent the means and standard errors (*n* = 5 plants). Asterisks indicate significant differences according to a Student’s *t*-test, ***P < 0.001. Rosette leaf samples collected in 3 biological repeats at 0, 2 and 4 h (arrows) were used for microarray analysis. Room temperature and relative room humidity were recorded during the dehydration period. (**B**) Diagrams illustrating experimental design and comparisons between the treatments. The number of differentially expressed genes (DEGs) identified from various comparisons are noted in red (upregulated relative to control) or blue (downregulated relative to control). Data were obtained from the microarray analysis of 3 biological repeats. (**C**) Venn diagram analysis showing the overlapping and non-overlapping DEGs among the comparisons. M-C/W-C, *kai2-2* well-watered control 0 h vs. WT well-watered control 0 h; M-D2/W-D2, *kai2-2* dehydrated 2 h vs. WT dehydrated 2 h; M-D4/W-D4, *kai2-2* dehydrated 4 h vs. WT dehydrated 4 h; M-D/W-D, M-D2/W-D2 and/or M-D4/W-D4; W-D2/W-C, WT dehydrated 2 h vs. WT well-watered control 0 h; W-D4/W-C, WT dehydrated 4 h vs. WT well-watered control 0 h; W-D/W-C, W-D2/W-C and/or W-D4/W-C; M-D2/M-C, *kai2-2* dehydrated 2 h vs. *kai2-2* well-watered control 0 h; M-D4/M-C, *kai2-2* dehydrated 4 h vs. *kai2-2* well-watered control 0 h; M-D/M-C, M-D2/M-C and/or M-D4/M-C.

To gain broader insights into how KAI2 signaling contributes to drought resistance, we performed transcriptomic profiling of *kai2* and WT leaves undergoing dehydration. A microarray analysis was conducted as illustrated in Fig 4A and 4B. The microarray data can be accessed through accession number GSE90622, and the results of the transcriptome analysis are summarized in S1 Table. A preset criteria of |fold change ≥ 2| and false discovery rate-corrected *P-*values (i.e. *q*-values) <0.05 was used to identify differentially expressed genes (DEGs) in each comparison (Fig 4B and S2 Table). The microarray data was validated by expression analysis of several selected genes using qRT-PCR (S2 Fig).

Under non-stressed, well-watered conditions, 680 transcripts were upregulated and 702 were downregulated in *kai2-2*, as indicated by the M-C/W-C (*kai2-2* control/WT control) comparison (Fig 4B and S2A–S2K Table). We compared the DEGs in well-watered *kai2-2* (M-C/W-C) to those we previously identified in well-watered *max2-3* [5]. We found 66 downregulated genes and 56 upregulated genes in common between *kai2-2* and *max2-3* plants (S3 Table). The downregulated genes included several prominent KAR-induced transcripts that were identified in prior studies, such as *DLK2* (*At3g24420*), *BZS1/STH7* (*At4g39070*), *KUF1* (*At1g31350*), *GA3ox1*, *SMAX1* (*At5g57710*), *SMXL2* (*At4g30350*), and *At3g60290* encoding a 2OG-Fe(II) oxidoreductase [11,23–25]. Consistent with our qRT-PCR analysis (Fig 3C), *CYP707A3* was also among this downregulated set of genes. Therefore, our analysis can provide reliable identification of genes that are regulated by *KAI2*.

We found that 216 (31.8%) of the upregulated transcripts and 366 (52.1%) of the downregulated transcripts in well-watered *kai2-2* plants were differentially expressed in a similar manner after 2 h and/or 4 h of dehydration (see comparison to M-D/W-D; Fig 4C(i) to 4C(ii) and S4A and S4B Table). Another 420 and 452 unique genes were respectively upregulated or downregulated in *kai2-2* relative to WT only after 2 h and/or 4 h dehydration, which may indicate genes whose regulation by KAI2 is only revealed under the drought condition (Fig 4C(i) and 4C(ii)). A high number of DEGs in *kai2-2* plants were also dehydration-responsive. Specifically, 29.9% and 43.8% of the genes downregulated in *kai2-2* under well-watered (M-C/W-C) or dehydration conditions (M-D/W-D), respectively, were induced by dehydration [Fig 4C(iii) and 4C(iv), and S4F and S4J Table]. Conversely, 31.9% to 33.7% of the genes upregulated in *kai2-2* in this experiment were repressed by dehydration [Fig 4C(v) and 4C(vi), and S4O and S4S Table].

### Transcriptome analysis of *kai2* mutant reveals anthocyanin and cuticle defects

We hypothesized that some of these transcriptional perturbations might reveal altered biochemical or physiological responses that contribute to the reduced drought resistance of the *kai2* plants. Therefore, we used MapMan to perform an in-depth survey of the functional categories of the DEGs identified in *kai2-2* plants (S2–S5 Figs). We found two gene categories to be of particular interest.

**Fig 5:**
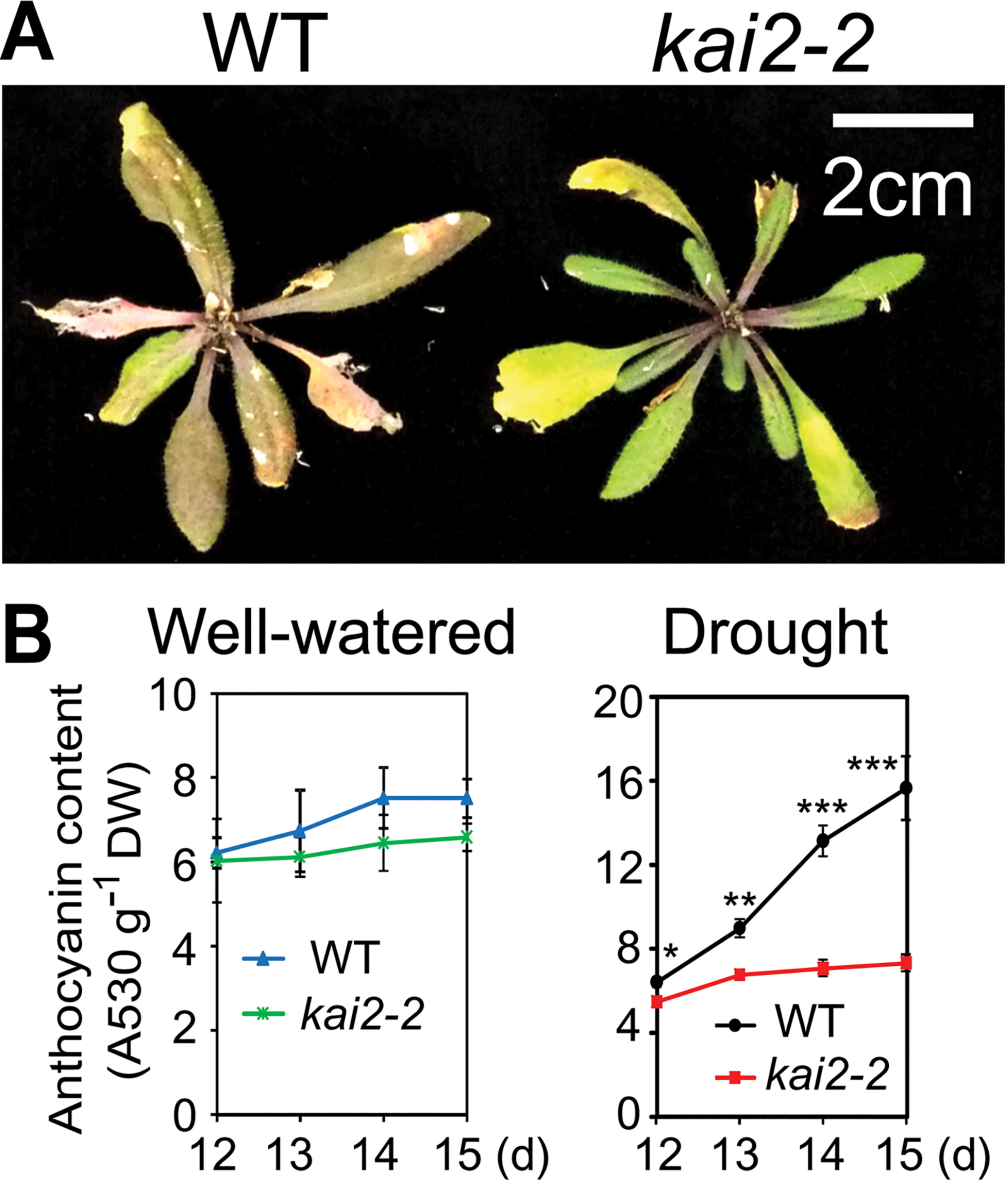
Anthocyanin production in *kai2-2* and WT plants. (**A**) *kai2-2* and WT plants were grown for 5 weeks, and watering was withheld for 10 days. Inflorescences were cut from representative plants before photographing. (**B**) Anthocyanin content in *kai2-2* and WT plants under well-watered and drought conditions. Data represent the means and standard errors (*n* = 4 plants). Asterisks indicate significant differences between the genotypes under drought conditions as determined by a Student’s *t*-test, *P < 0.05; **P < 0.01; ***P < 0.001.

First, we noted that many dehydration-inducible genes that positively regulate or carry out the synthesis of anthocyanins/flavonoids, such as *DFR*, *FLS1*, *F3’H*, *GL3*, *MYB75*/*PAP1* and *MYB90*/*PAP2* [26], were downregulated in *kai2-2* (S2–S4 Figs and S5A Table). By contrast, genes encoding negative regulators of anthocyanin biosynthesis, such as *MYBL2*, *LBD37*, and *LBD39* [26], were dehydration-repressible and upregulated in *kai2-2* under normal or dehydration conditions (S2–S4 Figs and S5A Table). Thus, we hypothesized that anthocyanin content might be lower in *kai2* plants than WT, particularly during water deficit. Because anthocyanins are known to provide protection to plants against drought [27], reduced anthocyanin content might contribute to the drought susceptibility of *kai2* plants. We observed that WT plants developed a darker leaf coloration than *kai2-2* mutants at late developmental stages, especially after water was withheld for seed maturation (Fig 5A). We also compared the anthocyanin contents in *kai2* and WT plants at various time points during a drought resistance assay. Anthocyanin levels were significantly lower in both *kai2-2* and *kai2-4* plants than WT under water deficit conditions (Fig 5B and S6A Fig).

**Fig 6:**
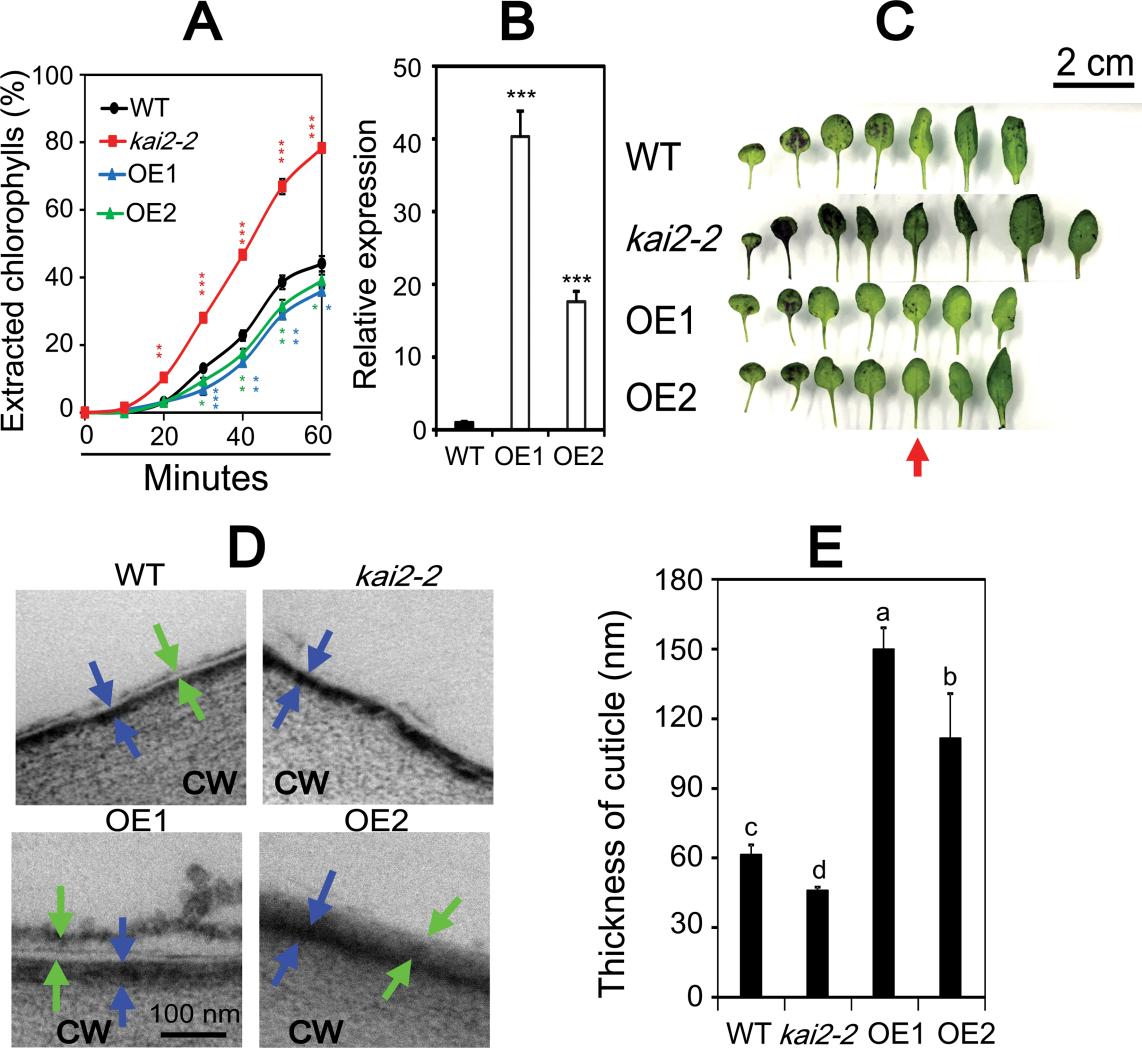
Cuticle permeability of *kai2-2*, *35S*:*KAI2* transgenic lines OE1 and OE2, and WT plants. (**A**) Chlorophyll leaching from rosette leaves of 28-day-old *kai2-2*, OE1, OE2 and WT plants at different time periods. Data represent the means and standard errors (*n* = 3 plants/genotype). (**B**) Fold-change of overexpression levels of *KAI2* gene in leaves of 14-day-old OE1 and OE2 plants in comparison with WT (*n* = 5 biological replicates). Asterisks indicate significant differences between the WT and other genotypes under well-watered condition as determined by a Student’s *t*-test, *P < 0.05; **P < 0.01; ***P < 0.001. (**C**) Toluidine blue staining patterns of rosette leaves of 28-day-old *kai2-2*, OE1, OE2 and WT plants. Red arrow indicates the fifth leaves, which were used for transmission electron microscope (TEM) analysis.(**D**) TEM images of the surface of the fifth leaves (adaxial side) derived from *kai2-2*, OE1, OE2 and WT plants. CW, cell wall. Blue arrows indicate cuticular layer (electron-dense, darker-staining layer) and green arrows indicate wax-rich cuticle proper (electron-translucent layer). (**E**) Thickness of cuticle of the fifth leaves (adaxial side) derived from *kai2-2*, OE1, OE2 and WT plants. Data represent the means and standard errors (*n* = 3 biological replicates). Different letters above the error bars indicate significant differences (*P* < 0.05) among the genotypes according to a Tukey's honest significant difference test.

Second, many genes involved in cuticle formation, such as *CER1*, *CER4*, *CYP96A15*, *MYB94*, *SHN1*/*WIN1*, *SHN2*, *SHN3* and *ABCG13* [4,28], were found to be dehydration-inducible and downregulated in unstressed and/or dehydrated *kai2-2* leaves (S2, S4, S5 Figs and S5B Table). We hypothesized that a cuticular defect could cause enhanced non-stomatal water loss that might explain the faster rate of RWC decline observed in drought-stressed *kai2* plants. To examine this possibility, we carried out a chlorophyll (Chl) leaching assay of rosette leaves (Fig 6A). We found that Chl leached much faster from leaves of both *kai2-2* and *kai2-4* than that of WT (Fig 6A and S6B Fig), suggesting that *kai2* mutants have higher cuticular water permeability than WT. In contrast, we noted lower Chl leaching rates from *35S*:*KAI2* overexpressor OE1 and OE2 plants than WT (Fig 6A and 6B), indicating that OE1 and OE2 plants have lower cuticular water permeability than WT. We also used toluidine blue (TB) staining to visualize potential defects in the leaf cuticle of *kai2* mutants. More leaves, especially the older leaves, of *kai2-2* and *kai2-4* mutants were stained compared with WT, whereas reduced staining was observed in the leaves of OE1 and OE2 relative to that of WT (Fig 6C and S6C Fig), supporting the results of the Chl leaching assay (Fig 6A and S6B Fig). Furthermore, we examined the surface of the fifth leaves of *kai2-2*, OE1, OE2 and WT plants with transmission electron microscopy (TEM). A cuticle proper, which is a layer of lipid polymer filled with wax [29], was detected in leaves of WT, OE1 and OE2, but not in *kai2-2* leaves (Fig 6D), revealing a significant structural defect that is likely to contribute to faster water loss in *kai2* plants. Additionally, *kai2-2* mutant plants were found to have thinner cuticles than WT, whereas OE1 and OE2 lines have thicker cuticles (Fig 6E).

### D14 also contributes to drought resistance

The role of SLs in drought resistance of *Arabidopsis* has been unclear. Bu et al. (2014) reported that *max2* mutants have defects in drought survival, higher water loss from detached leaves, and decreased germinative greening under stress or ABA treatments, but SL-deficient *max* mutant plants resemble WT [6]. In contrast, Ha et al. (2014) showed reduced drought resistance and ABA hyposensitivity in *max2* and SL-deficient *max* mutants [5]. Supporting this, SL-depleted tomato and *L*. *japonicus* have reduced resistance to drought and osmotic stress, respectively [7,8]. These conflicting observations may be reconcilable if SLs are only responsible for some of MAX2-dependent drought resistance.

**Fig 7:**
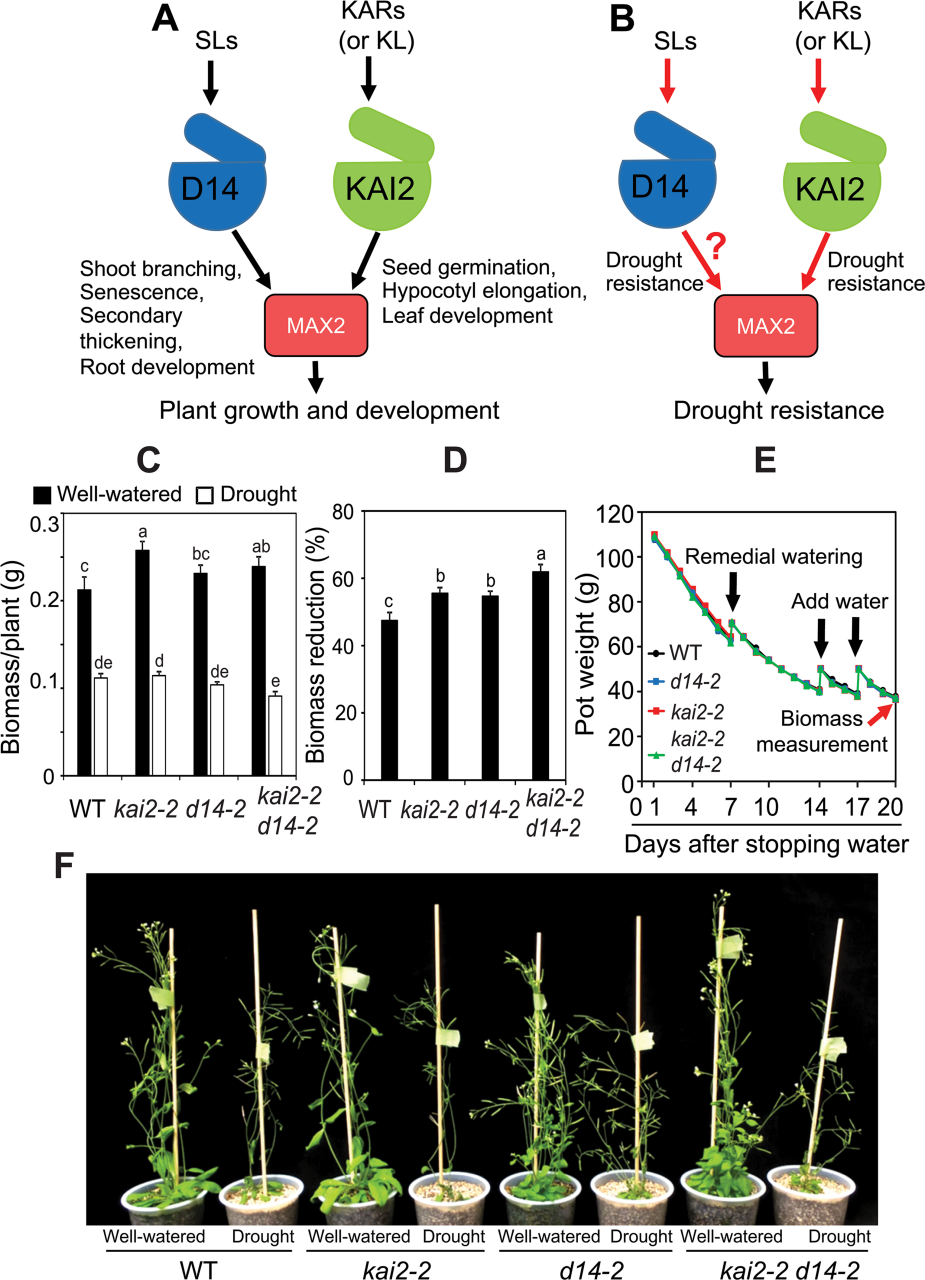
Models for functions of SLs and KARs/KAI2-ligand (KL) in plant growth, development and drought response. (**A**) SL-regulation of shoot branching, senescence, secondary thickening and root development is mediated by SL-specific receptor D14. KAR-regulation (or hypothetical KL-regulation) of seed germination, hypocotyl elongation and leaf development is mediated by KAI2. MAX2 is the checkpoint for both SL and KAR/KL signaling pathways in plant growth and development. (**B**) SLs and KARs/KL regulate plant resistance to drought through D14-MAX2 and KAI2-MAX2 cascade, respectively. Question mark indicates the contribution of D14 to drought resistance was unknown until this study. (**C**) Biomass of *kai2-2*, *d14-2*, *kai2-2 d14-2* and WT plants under well-watered control and drought stress (*n* = 9 biological replicates). (**D**) Biomass reduction of *kai2-2*, *d14-2*, *kai2-2 d14-2* and WT plants under drought relative to respective well-watered control. Data represent the means and standard errors (*n* = 9 biological replicates). Different letters above the error bars indicate significant differences (*P* < 0.05) among the genotypes according to a Tukey's honest significant difference test. (**E**) Averaged pot weights of *kai2-2*, *d14-2*, *kai2-2 d14-2* and WT plants during drought stress (*n* = 9 biological replicates). Black arrows indicate when water was added to the root growth area in the soil. Red arrow indicates when biomass was measured. (**F**) Plant phenotypes before harvest.

To date, all SCF^MAX2^-dependent SL responses in plant growth and development have been shown to be mediated by the SL receptor D14 (Fig 7A). Because our experiments showed that KAI2 is involved in drought resistance, it raised the question of whether D14 also participates in this response (Fig 7B). Thus, we analyzed drought resistance of the *d14-2* allele [30]. We found that *d14-2* had a higher reduction in biomass than WT in the gravimetric method-based drought-resistance assay (Fig 7C–7F). Therefore, D14 acts as a positive regulator of drought resistance. In addition, we noted that *kai2-2 d14-2* double mutants were less able to accumulate biomass under water restrictions than either of the single mutants (Fig 7C–7F), suggesting that both D14- and KAI2-mediated signaling pathways may act together, perhaps through MAX2 [9,11,12], to enhance drought resistance in *Arabidopsis*. Interestingly, we did not observe cuticle defect in *d14-2* leaves like in that of *kai2-2* single and *kai2-2 d14-2* double mutants (S7 Fig), suggesting that drought-sensitive phenotype of *d14* plants is not associated with defect in cuticle development.

## Discussion

Although KARs and SLs typically have distinct effects on plant growth and development, these signals are perceived by similar mechanisms that require MAX2 [12,31]. Several studies previously demonstrated that MAX2 and SLs have positive regulatory roles in plant adaptation to drought [5–8]; but possible contributions of KARs or KL had not been examined. In this study, we aimed to investigate the roles of KAI2-dependent signaling in plant response to drought using in-depth physiological, biochemical and molecular characterization of the loss-of-function *kai2* mutants. We implicated *KAI2*-dependent signaling in plant response to drought as a positive regulator.

Our analyses of the *kai2* mutants provided evidence that its reduced biomass under drought was associated with an inability to maintain high leaf RWC during water deficit (Fig 1B, Fig 2A and 2B, S1B Fig). A higher transpiration rate, which increases water loss, was suggested by remarkably lower leaf temperatures in *kai2* plants than in WT under both well-watered and water-deficit conditions (Fig 2I and S1E Fig). We identified several ways in which KAI2-dependent signaling likely contributes to drought resistance. First, it is well-established that cell membrane stability, reflected by the levels of cellular electrolyte leakage, is a major factor contributing to the maintenance of water status in plants during water deficit [32,33]. Thus, the increase in electrolyte leakage linked with reduced RWC recorded in the *kai2* mutants during drought (Fig 2A–2D and S1A–S1D Fig) suggested that *kai2* suffered a severe stress-induced cell membrane damage. This might in part result from a decrease in reactive oxygen species (ROS)-scavenging antioxidants [33], as supported by the observed reduction in endogenous anthocyanin levels (Fig 5B and S6A Fig), ultimately leading to a higher rate of water loss.

Second, a reduction in ABA-regulated stomatal closure in *kai2* plants, which may contribute to increased water loss (Fig 2F–2H), might enhance drought susceptibility. The impaired stomatal movement in *kai2* mutants was in good agreement with the strong downregulation of *ABCG40*/*AT1G15520* and *ABCG22*/*AT5G06530* in both unstressed and stressed *kai2* plants (12.1- and 17.7-fold for *ABCG40*, and 3.92- and 1.79-fold for *ABCG22*, respectively, in normal and dehydrated *kai2* vs WT) (S1 Table). *ABCG40* is a key ABA transporter, and *ABCG22* putatively has a similar function; these genes were shown to regulate stomatal closure and drought resistance in *Arabidopsis* [34,35]. The *abcg40* mutant has inefficient ABA-mediated stomatal closure [34], whereas *abcg22* shows a defect in stomatal closing that is perhaps dependent on ABA signaling [35]. Both *abcg22* and *abcg40* mutants exhibited drought-susceptible phenotypes, further supporting the link [34,35]. Notably, previous studies reported that the drought-susceptible *max2* plants also have impaired ABA-mediated stomatal closure [5,6], and exhibit downregulation of *ABCG22* and *ABCG40* under both normal and dehydration conditions [5]. Furthermore, the impairment of ABA-mediated stomatal closure and increased cotyledon opening rates of *kai2* mutants treated with exogenous ABA (Figs 2F–2H and 3A, S1F Fig) indicate hyposensitive responses to ABA. This finding suggests that crosstalk between ABA- and KAI2-dependent signaling pathways may influence plant adaptation to drought. We noted an increase in ABA content in *kai2* leaves during dehydration (Fig 3B), which might be attributed to the downregulation of the key ABA catabolic enzyme *CYP707A3* (Fig 3C and S2 Fig). This result suggests that *kai2* mutants may produce higher levels of ABA to compensate for its reduced ability to respond to ABA; such a feedback mechanism may attenuate the severity of the *kai2* drought-resistant phenotype.

Third, non-stomatal water evaporation associated with higher cuticular permeability is likely to increase the rate of RWC decline of *kai2* plants under drought [28]. Unlike in leaves of WT, in leaves of *kai2* plants we did not detect a cuticle proper (Fig 6D). Additionally, we identified thinner cuticles in leaves of *kai2* than that of WT by using TEM analysis (Fig 6D and 6E), and observed higher permeability through a Chl leaching assay and TB staining (Fig 6A and 6C, S6B–S6C Fig). At a molecular level, our comparative transcriptome analysis suggested that the altered structure of the *kai2* cuticle may be due to downregulation of several genes involved in the biosynthesis and transport of wax, such as *CER1*, *CYP96A15*, *WSD1*, *MYB94*, *MYB16*, *SHN1*, *SHN2* and *SHN3* (S3–S6 Figs) [4,36]. Some of the *KAI2*-regulated cuticle formation-related genes, such as the *MYB16* and *MYB94* transcription factors, are also controlled by ABA [4]. Similar to our observations, the drought-sensitive *max2* mutant plants were found to have a defect in cuticular architecture [6], which might be attributed to downregulation of genes involved in cuticle formation as well (S5B Table) [5]. Additionally, loss of *KAI2* also downregulated the expression of cutin biosynthesis-related genes (S5 Fig). For example, *CED1*/*At1g64670* [37,38], which minimizes water loss through not only by enhancing cuticle structure but also by mediating ABA and osmotic stress signaling [39], was downregulated in *kai2* versus WT after 4 h dehydration (S5 Fig). These findings support the positive role of KAI2 in mediating drought resistance, and the link among cuticle formation, KAI2 signaling, ABA signaling and osmotic stress responses in plants.

Fourth, the decline in anthocyanin production in *kai2* plants during drought may contribute to its enhanced drought sensitivity. This finding is supported by a number of studies that have found a positive correlation between drought resistance and anthocyanin levels in *Arabidopsis* as the ROS-scavenging antioxidant function of anthocyanins can protect cells from drought [27,40]. The reduced anthocyanin accumulation in *kai2* plants (Fig 5B and S6A Fig) may be explained by transcriptional misregulation of the anthocyanin biosynthetic pathway (S2–S4 Figs). In agreement with this idea, several anthocyanin/flavonoid biosynthesis-related genes were found to be upregulated in KAR_1_-treated *Arabidopsis* seeds, and downregulated in *kai2* seedlings grown under different light conditions, leading to reduced accumulation of anthocyanin pigments [24,41]. Similarly, many anthocyanin biosynthesis-related genes were shown to be downregulated in *max2* plants under dehydration conditions (S5A Table) [5]. Furthermore, a quantitative proteomic analysis of *max2* seedlings identified a set of proteins involved in flavonoid biosynthesis that have reduced abundance relative to WT; a subset of these proteins are induced by *rac*-GR24 treatment [42]. Both purified enantiomers of GR24 were able to stimulate flavonol production, and importantly, *rac*-GR24 enhanced flavonol accumulation in both *d14* and *kai2* mutants [42]. Therefore, there is ample evidence to support regulation of anthocyanin and flavonoid syntheses by both MAX2-dependent D14 and KAI2 signaling pathways. Thus, we propose that accumulation of anthocyanin under drought might be an important aspect of stress resistance conferred by KAI2. Altogether these observations support the involvement of a KAR/KL-KAI2-SCF^MAX2^ signaling cascade in regulating plant drought adaptation through controlling cell membrane stability, stomatal movement, cuticle development and anthocyanin production.

**Fig 8:**
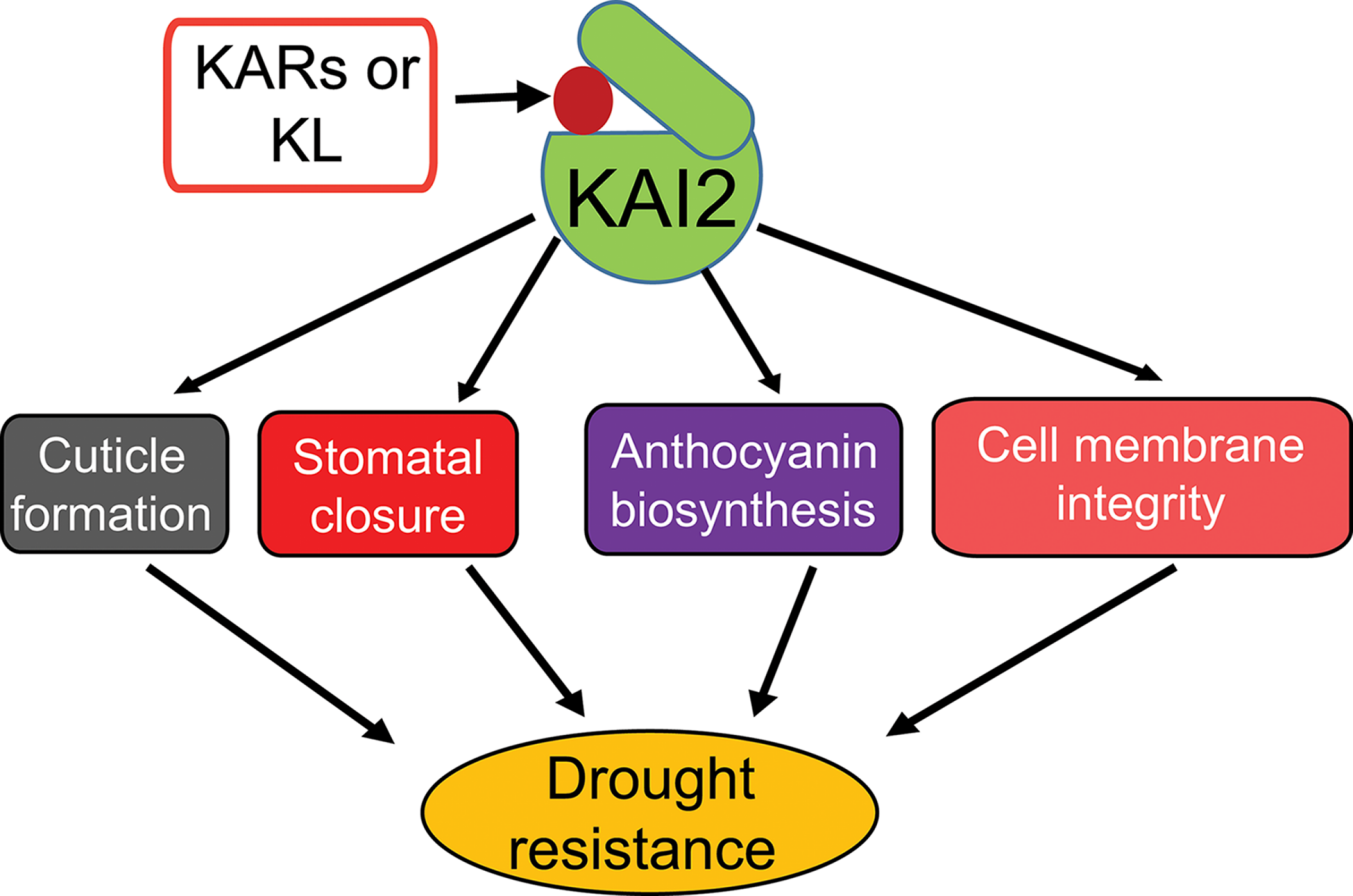
Model illustrating functions of KAI2 in plant resistance to drought. Karrikins (KARs) or a putative, endogenous KAI2 ligand (KL) activate KAI2 signaling, which promotes plant resistance to drought through several biochemical and physiological processes.

In summary, our results demonstrate that KAI2-mediated signaling positively regulates drought resistance in *Arabidopsis*. KAI2 is activated through the binding of KARs (or as-yet-unknown KL), resulting in the regulation of downstream genes involved in cuticle formation, stomatal closure, anthocyanin biosynthesis and membrane integrity. These adjustments may collectively contribute to plant adaptation to drought (Fig 8). Importantly, our results suggest that genetic manipulation to enhance *KAI2* and *D14* signaling pathways, either alone or together, is a promising avenue for the improvement of crop productivity in arid lands.

## Materials and methods

### Plant materials

*Arabidopsis thaliana* Columbia ecotype (Col-0) was used as WT in all experiments. The *kai2-2* (SGT6839) and *kai2-4* (GT6185) alleles were obtained from the *Arabidopsis* Biological Resource Center [11,43]. The *kai2-2* and *kai2-4* mutants were originally in the Ler background and were backcrossed with Col-0 six times [43]. The *d14-2* mutant was obtained from the TILLING project (http://tilling.fhcrc.org/) after backcrossing twice with Col-0 [30]. The *kai2-2 d14-2* double mutant was made by crossing the *d14-2* and *kai2-2* mutants. To make *KAI2*-overexpressing plants, the *KAI2* cDNA was amplified by PCR (primers listed in S6 Table), and cloned into the pGWB2 expression vector under the control of the CaMV35S promoter [44]. The resulting *35S*:*KAI2* plasmid was introduced into WT (Col-0) plants using *Agrobacterium tumefaciens*-mediated transformation method [45]. Two homozygous *35S*:*KAI2* transgenic lines OE1 and OE2 with a single transgene copy obtained by selection for three consecutive generations were used in the study.

### Drought resistance assayes

Drought-responsive phenotypes were examined using the gravimetric method described by Harb and Pereira [20]. Briefly, 2-week-old plants grown on germination medium (GM) were transferred to plastic pots (7×7 cm in diameter and height) containing 28.7 g dry soil (Dio Propagation Mix No.2 for Professional; Dio Chemicals Ltd.). Plants were then grown on pots saturated with water for 10 days before they were exposed to drought stress. After watering was stopped for 7 days, remedial watering of several pots was conducted to ensure that all pots had almost the same amount of soil water content. Seven days after remedial watering, each pot received a suitable amount of water to reach the weight of 60 g. The pots were then dried for 3 days; thereafter, they received a suitable volume of water to again reach the weight of 60 g. This process was repeated two times so that each pot had similar soil water content during drying. The weight of the pots was measured every day during the experiment. Twenty days after drought stress, whole aerial parts of plants were cut and packed in paper bags. The well-watered plants were also harvested at the same time. The bags were then oven-dried at 65°C for two days, and the dry weight (biomass) of aerial parts of each plant was measured. The biomass reduction was calculated using the following equation:

Biomassreduction(%)=[(Dryweightofwell-wateredplant-Dryweightofstressedplant)×100]/(Dryweightofwell-wateredplant).

In addition, we also adapted the method of Bu et al. (2014) [6] to compare the drought resistance of the *kai2* mutant and WT plants grown in high density. Seeds of mutant and WT plants (50 seeds/each/pot) were sown directly side-by-side in the same pots (7×7 cm in diameter and height) containing soil (Dio Propagation Mix No.2 for Professional; Dio Chemicals Ltd.) saturated with water. Eight days after sowing, water was withheld from plants for 14 days. Photographs were taken at days 7^th^ (15-day-old plants) and 14^th^ (22-day-old plants) during the drought assay.

### RWC and electrolyte leakage of plants exposed to drought stress

RWC and electrolyte leakage of the detached aerial parts of different genotypes exposed to drought stress were determined according to Nishiyama et al. (2011) [46]. Briefly, two-week-old mutants and WT plants grown on GM were transferred side-by-side (30 plants for each phenotype) to trays (21×30×5cm in width, length and height) containing soil (Dio Propagation Mix No.2 for Professional; Dio Chemicals Ltd.) saturated with water. After one week of transfer, water irrigation was stopped to induce drought stress. During the assay, relative soil moisture content of both sides of the trays was followed using a HydroSense soil moisture probe (Campbell Scientific Inc.) as previously described [46]. Shoots of well-watered and drought-stressed plants (*n* = 4/genotype) were harvested every day from day 11 to day 15 for determination of RWC using the procedure adapted from Barrs and Weatherley [47]. Briefly, detached shoot samples were weighed to determine the weight (W) of each individual, and the samples were then placed into 50-mL tubes containing 40 mL of deionized water for 3-hour rehydration to full turgidity under room temperature. After rehydration, water was gently removed from shoot samples by filter paper, and the turgid weight (TW) of each sample was determined. Subsequently, the shoot samples were put into paper bags, oven-dried (65°C) for 48 h, and then dry weight (DW) of each oven-dried sample was measured. RWC was calculated by using the following equation: RWC (%) = [(W-DW)/(TW-DW)] × 100. Detached aerial parts of different genotypes (*n* = 4/genotype) exposed to drought stress as described above were also harvested for determination of electrolyte leakage as previously described by Nishiyama et al. (2011) [46].

### Stomatal aperture and density

Stomatal aperture and stomatal density were measured as previously described [5], with a slight modification for stomatal aperture closure assay. Epidermal peels from leaves of 21-day-old plants grown on GM plates were incubated in a solution containing 0.2 mM CaCl_2_, 10 mM KCl, and 10 mM Mes·KOH (pH 6.15) under white light (300 μmol·m^−2^·s^−1^) for 12 h. Thereafter, the samples were incubated in the same buffer solution containing different concentrations of ABA for 1 h before the stomatal aperture was measured.

### Leaf surface temperature and quantification of ABA

Surface temperature of leaves was determined by using a thermal video system (TVS-8500; Nippon Avionics) [48]. Quantification of ABA in leaves of 24-day-old WT and *kai2-2* plants grown on soil, which were detached and exposed to dehydration according to published method [5], was performed as previously described [49].

### Cotyledon opening assay

Cotyledon opening assay was used as a means to assess the ABA sensitivity of various genotypes. Seeds were sowed on GM containing 1% sucrose and various concentrations of ABA, and opened cotyledons were counted according to published method [46].

### Anthocyanin content

WT and mutant plants were grown on the same tray and subjected to a drought treatment as described above. At indicated time points, aerial parts (without inflorescence) of stressed and well-watered plants were separately collected, and frozen dry weight and anthocyanin content were measured [50].

### Chl leaching assay and TB staining

Chlorophyll leaching assays were performed as described previously [6]. Briefly, the aerial parts (without inflorescence) of 4-week-old plants were incubated on ice for 30 min, and then immersed in 40 mL 80% ethanol (v/v) at room temperature. Solution samples (100 μL) were taken every 10 min after immersion to quantify the amount of chlorophyll content. TB staining was used to detect cuticular defects on leaves [51]. Aerial parts (without inflorescence) of 4-week-old plants were submerged into a solution of 0.05% (w/v) TB for 2 h. Plants were then gently transferred to water and softly shaken to remove excessive TB. Rosette leaves were separated and placed on dry paper for taking photos.

### TEM analysis

For cuticle observation, the fifth rosette leaves from 4-week-old *kai2-2*, *d14-2*, *kai2-2 d14-2*, *35S*:*KAI2* (OE1, OE2), and WT plants were analyzed as described previously [52], with a slight modification. Briefly, the top part of the leaves (5 mm) was cut into 1 × 3 mm rectangles and fixed with 4% paraformaldehyde and 2% glutaraldehyde in 50 mM sodium cacodylate buffer (pH 7.4) overnight at 4°C. The ultrathin sections were observed with a JEOL JEM-1400 TEM at 80 kV. Detailed observation and analysis using TEM were performed according to Toyooka et al. (2000) [53]. The thickness of the cuticle was measured using ImageJ software (https://imagej.nih.gov/ij/index.html).

### Dehydration treatment of soil-grown seedlings and transcriptome analysis

Aerial rossette leaves of 24-day-old WT and *kai2-2* plants grown on soil under well-watered conditions were detached and exposed to dehydration for the indicated time periods for determination of RWC and sample collection as previously described [5]. RWC of dehydrated shoot samples was determined following the same method adapted from Barrs and Weatherley [47] described above. Leaves of WT and *kai2-2* plants treated by dehydration for 0, 2 and 4 h were collected in 3 biological repeats for transcriptome analysis using the *Arabidopsis* Oligo 44K DNA microarray (Version 4.0, Agilent Technology) [54]. The criteria of |fold-change ≥ 2| and a false discovery rate corrected p-value (q-value) <0.05 were used in identifying the DEGs [55]. The raw microarray data and detailed protocol were deposited in the Gene Expression Omnibus database (GSE90622). When necessary, MapMan (http://mapman.gabipd.org), or *Arabidopsis* eFP browser (http://bar.utoronto.ca/efp_arabidopsis/cgi-bin/efpWeb.cgi) were used to analyze the data.

### qRT-PCR analysis

Total RNA was extracted using TRIzol Reagent (Invitrogen). Previously described procedures were used for cDNA synthesis and qRT-PCR analysis [56], in which *UBQ10* was used as a reference gene. Primer pairs used in qRT-PCR are listed in S6 Table.

## Supporting information

S1 Fig Drought-associated traits of leaves and ABA-response of cotyledon opening in *kai2-4* and WT. (**A-C**) *kai2-4* and WT plants were grown and exposed to drought. At indicated time points, (**A**) soil relative moisture contents (*n* = 10) and relative humidity, (**B**) leaf relative water content (RWC) (*n* = 4 biological replicates), and (**C**) electrolyte leakage (*n* = 4 biological replicates) were determined. (**D**) Electrolyte leakage (*Left*) of *kai2-4* and WT plants at a similar RWC (*Right*) during drought treatment (*n* = 4 biological replicates). (**E**) Leaf surface temperature of well-watered (21-day-old) *kai2-4* and WT plants. Thermal imaging camera (Top) and common optical camera (Bottom) were used to take pictures at the same time. (**F**) Cotyledon opening percentage of *kai2-4* and WT seeds in the absence or presence of different concentrations of exogenous ABA. Data represent the means and standard errors of 3 independent experiments (*n* = 50 seeds/genotype/experiment). Asterisks indicate significant differences as determined by a Student’s *t*-test, *P < 0.05; **P < 0.01; ***P < 0.001.
(TIF)

S2 Fig Verification of microarray data by qRT-PCR. (**A**) Heatmap presentation indicates the fold-changes in expression of representative genes derived from microarray analysis. (**B**) Heatmap presentation indicates the fold-changes in expression of representative genes using qRT-PCR. Expression data were obtained from microarray analysis or qRT-PCR of 24-day-old *Arabidopsis* rosette leaf samples that were collected from 3 independent plants for microarray analysis (*n* = 3). *UBQ10* was used as reference gene in qRT-PCR analysis. Relative expression levels are indicated by intensities of colors expressed in fold-change with saturation at 6. Red and blue colors indicate up- and downregulation, respectively. Note that not all data points shown in (a) passed the q-value < 0.05. M-C, *kai2-2* well-watered control; M-D2, *kai2-2* dehydrated 2 h; M-D4, *kai2-2* dehydrated 4 h; W-C, WT well-watered control; W-D2, WT dehydrated 2 h; W-D4, WT dehydrated 4 h.
(TIF)

S3 Fig Expression levels of anthocyanin biosynthesis-related genes in *kai2-2* and WT plants under well-watered and dehydration conditions. Heatmap presentation indicates the fold-changes in gene expression derived from microarray data. Relative expression levels are indicated by intensities of colors expressed in fold-change with saturation at 6. Red and blue colors indicate up- and downregulation, respectively. Note that not all data points shown passed the q-value < 0.05. M-C, *kai2-2* well-watered control; M-D2, *kai2-2* dehydrated 2 h; M-D4, *kai2-2* dehydrated 4 h; W-C, WT well-watered control; W-D2, WT dehydrated 2 h; W-D4, WT dehydrated 4 h.
(TIF)

S4 Fig MapMan analysis illustrates secondary metabolism-associated genes differentially expressed in M-C/W-C and/or M-D/W-D comparisons. (**A**) Downregulated genes in M-C/W-C and/or M-D/W-D. (**B**) Upregulated genes in M-C/W-C and/or M-D/W-D. Green and red colors show down- and upregulation, respectively. Colored bars in each panel indicate fold-changes in gene expression. M-C/W-C, *kai2-2* well-watered control versus WT well-watered control; M-D/W-D represents M-D2/W-D2 and/or M-D4/W-D4; M-C, *kai2-2* well-watered control; M-D2, *kai2-2* dehydrated 2 h; M-D4, *kai2-2* dehydrated 4 h; W-C, WT well-watered control; W-D2, WT dehydrated 2 h; W-D4, WT dehydrated 4 h. For repetitive genes, their highest fold-change was used in the analysis.
(TIF)

S5 Fig Expression levels of cuticle formation-related genes in *kai2-2* and WT plants under well-watered and dehydration conditions. Heatmap presentation indicates the fold-changes in gene expression derived from microarray data. Relative expression levels are indicated by intensities of colors expressed in fold-change with saturation at 6. Red and blue colors indicate up- and downregulation, respectively. Note that not all data points shown passed the q-value < 0.05. M-C, *kai2-2* well-watered control; M-D2, *kai2-2* dehydrated 2 h; M-D4, *kai2-2* dehydrated 4 h; W-C, WT well-watered control; W-D2, WT dehydrated 2 h; W-D4, WT dehydrated 4 h.
(TIF)

S6 Fig Anthocyanin production and cuticle permeability in *kai2-4* and WT. (**A**) Anthocyanin content in *kai2-4* and WT plants under drought conditions. Data represent the means and standard errors (*n* = 4 plants). (**B**) Chlorophyll leaching from rosette leaves of 28-day-old *kai2-4* and WT plants at different time periods. Data represent the means and standard errors (*n* = 3 plants/genotype). (**C**) Toluidine blue staining patterns of rosette leaves of 28-day-old *kai2-4* and WT plants. Asterisks indicate significant differences as determined by a Student’s *t*-test, *P < 0.05; **P < 0.01; ***P < 0.001.
(TIF)

S7 Fig Thickness of leaf cuticle of *kai2-2*, *d14-2*, *kai2-2 d14-2* and WT plants. (**A**) Thickness of cuticle of the fifth leaves (adaxial side) derived from *kai2-2*, *d14-2*, *kai2-2 d14-2* and WT plants. Blue arrows indicate cuticular layer (electron-dense, darker-staining layer) and green arrows indicate wax-rich cuticle proper (electron-translucent layer). (**B**) Transmission electron microscope images of the surface of the fifth leaves (adaxial side) derived from *kai2-2*, *d14-2*, *kai2-2 d14-2* and WT plants. CW, cell wall. Data represent the means and standard errors (*n* = 3 biological replicates). Different letters above the error bars indicate significant differences (*P* < 0.05) among the genotypes according to a Tukey's honest significant difference test.
(TIF)

S1 Table Comparative microarray analysis of leaves of *kai2-2* and WT plants under well-watered and dehydration conditions. (XLS)

S2 Table List of differentially expressed genes with at least twofold change in various comparisons. (**A**) List of upregulated genes in the M-C/W-C comparison. (**B**) List of upregulated genes in the M-D2/W-D2 comparison. (**C**) List of upregulated genes in the M-D4/W-D4 comparison. (**D**) List of upregulated genes in the M-D/W-D (e.g. in M-D2/W-C and/or M-D4/W-C) comparison. (**E**) List of upregulated genes in the W-D2/W-C comparison. (**F**) List of upregulated genes in the W-D4/W-C comparison. (**G**) List of upregulated genes in the W-D/W-C (e.g. in W-D2/W-C and/or W-D4/W-C) comparison. (**H**) List of upregulated genes in the M-D2/M-C comparison. (**I**) List of upregulated genes in the M-D4/M-C comparison. (**J**) List of upregulated genes in the M-D/M-C (e.g. in M-D2/M-C and/or M-D4/M-C) comparison. (**K**) List of downregulated genes in the M-C/W-C comparison. (**L**) List of downregulated genes in the M-D2/W-D2 comparison. (**M**) List of downregulated genes in the M-D4/W-D4 comparison. (**N**) List of downregulated genes in the M-D/W-D (e.g. in M-D2/W-D2 and/or M-D4/W-D4) comparison. (**O**) List of downregulated genes in the W-D2/W-C comparison. (**P**) List of downregulated genes in the W-D4/W-C comparison. (**Q**) List of downregulated genes in the W-D/W-C (e.g. in W-D2/W-C and/or W-D4/W-C) comparison. (**R**) List of downregulated genes in the M-D2/M-C comparison. (**S**) List of downregulated genes in the M-D4/M-C comparison. (**T**) List of downregulated genes in the M-D/M-C (e.g. in M-D2/M-C and/or M-D4/M-C) comparison. M-C, *kai2-2* well-watered control; M-D2, *kai2-2* dehydrated 2 h; M-D4, *kai2-2* dehydrated 4 h; W-C, WT well-watered control; W-D2, WT dehydrated 2 h; W-D4, WT dehydrated 4 h.
(XLS)

S3 Table Differentially expressed genes with at least twofold change in both *kai2-2* vs. WT and *max2-3* vs. WT comparisons under well-watered conditions. (**A**) List of upregulated genes. (**B**) List of downregulated genes.
(XLS)

S4 Table Venn analysis of differentially expressed gene sets derived from various comparisons. (**A**) Genes upregulated in M-C/W-C and M-D/W-D. (**B**) Genes downregulated in M-C/W-C and M-D/W-D. (**C**) Genes upregulated in W-D/W-C and M-D/M-C. (**D**) Genes downregulated in M-C/W-C and upregulated in W-D/W-C. (**E**) Genes downregulated in M-C/W-C and upregulated in M-D/M-C. (**F**) Genes downregulated in M-C/W-C and upregulated in W-D/W-C and/or M-D/M-C. (**G**) Genes downregulated in M-C/W-C and upregulated in both W-D/W-C and M-D/M-C. (**H**) Genes downregulated in M-D/W-D and upregulated in W-D/W-C. (**I**) Genes downregulated in M-D/W-D and upregulated in M-D/M-C. (**J**) Genes downregulated in M-D/W-D and upregulated in W-D/W-C and/or M-D/M-C. (**K**) Genes downregulated in M-D/W-D and upregulated in both W-D/W-C and M-D/M-C. (**L**) Genes downregulated in W-D/W-C and M-D/M-C. (**M**) Genes upregulated in M-C/W-C and downregulated in W-D/W-C. (**N**) Genes upregulated in M-C/W-C and downregulated in M-D/M-C. (**O**) Genes upregulated in M-C/W-C and downregulated in W-D/W-C and/or M-D/M-C. (**P**) Genes upregulated in M-C/W-C and downregulated in both W-D/W-C and M-D/M-C. (**Q**) Genes upregulated in M-D/W-D and downregulated in W-D/W-C. (**R**) Genes upregulated in M-D/W-D and downregulated in M-D/M-C. (**S**) Genes upregulated in M-D/W-D and downregulated in W-D/W-C and/or M-D/M-C. (**T**) Genes upregulated in M-D/W-D and downregulated in both W-D/W-C and M-D/M-C. M-C, *kai2-2* well-watered control; M-D2, *kai2-2* dehydrated 2 h; M-D4, *kai2-2* dehydrated 4 h; W-C, WT well-watered control; W-D2, WT dehydrated 2 h; W-D4, WT dehydrated 4 h.
(XLS)

S5 Table List of differentially expressed genes (DEGs) related to anthocyanin biosynthesis and cuticle formation. (**A**) List of DEGs related to anthocyanin biosynthesis. (**B**) List of DEGs related to cuticle formation.
(XLS)

S6 Table Primers used in qRT-PCR and *KAI2* cloning. (XLS)
