# Supplementary figures and images for: The karrikin receptor KAI2 promotes drought resistance in Arabidopsis thaliana
Source: PLoS Genet. 2017 Nov 13;13(11):e1007076. doi: 10.1371/journal.pgen.1007076 (PMC5703579; doi:10.1371/journal.pgen.1007076)

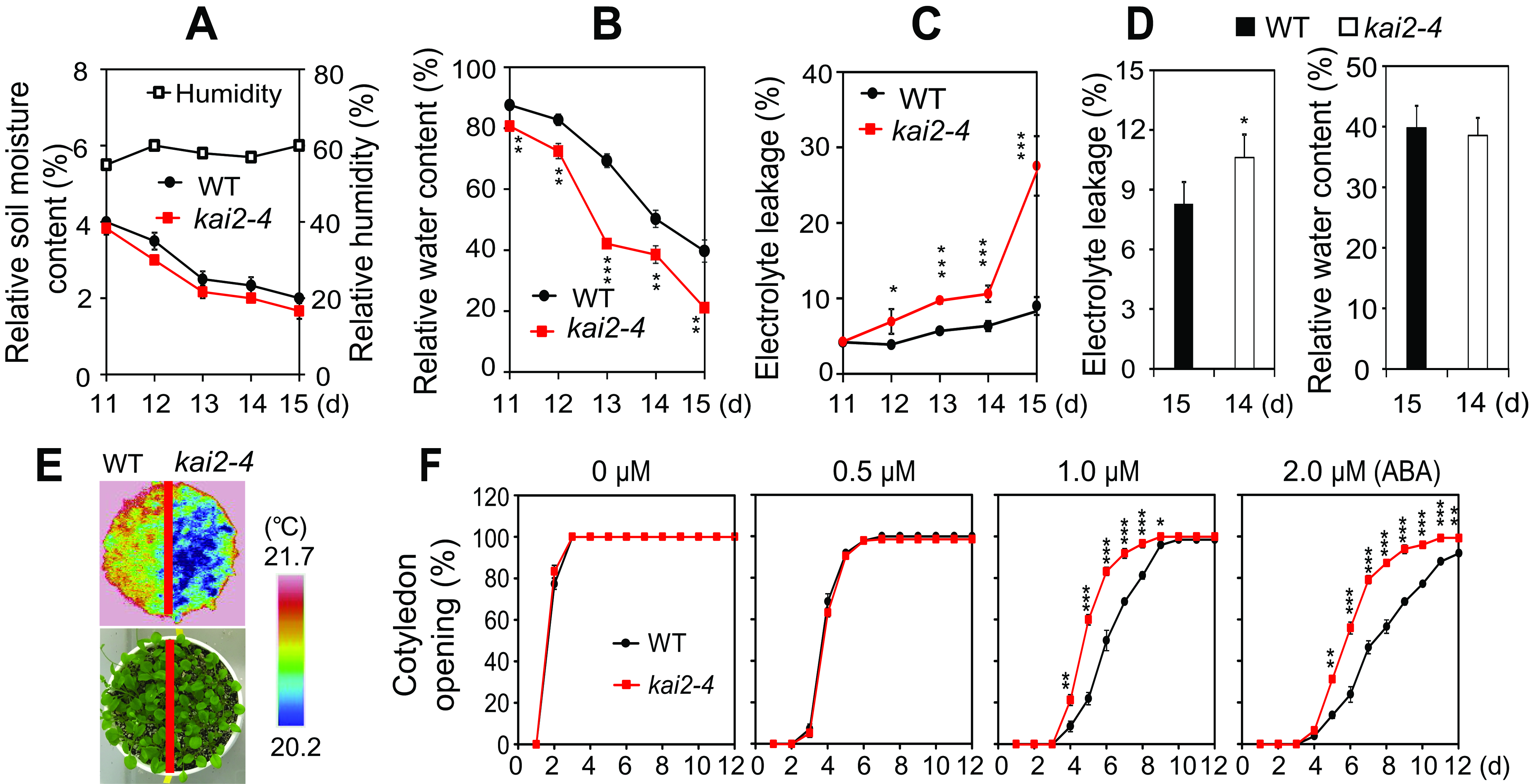

Supplement: S1 Fig — (A-C) kai2-4 and WT plants were grown and exposed to drought. At indicated time points, (A) soil relative moisture contents (n = 10) and relative humidity, (B) leaf relative water content (RWC) (n = 4 biological replicates), and (C) electrolyte leakage (n = 4 biological replicates) were determined. (D) Electrolyte leakage (Left) of kai2-4 and WT plants at a similar RWC (Right) during drought treatment (n = 4 biological replicates). (E) Leaf surface temperature of well-watered (21-day-old) kai2-4 and WT plants. Thermal imaging camera (Top) and common optical camera (Bottom) were used to take pictures at the same time. (F) Cotyledon opening percentage of kai2-4 and WT seeds in the absence or presence of different concentrations of exogenous ABA. Data represent the means and standard errors of 3 independent experiments (n = 50 seeds/genotype/experiment). Asterisks indicate significant differences as determined by a Student’s t-test, *P < 0.05; **P < 0.01; ***P < 0.001. (TIF) [file pgen.1007076.s001.tif]

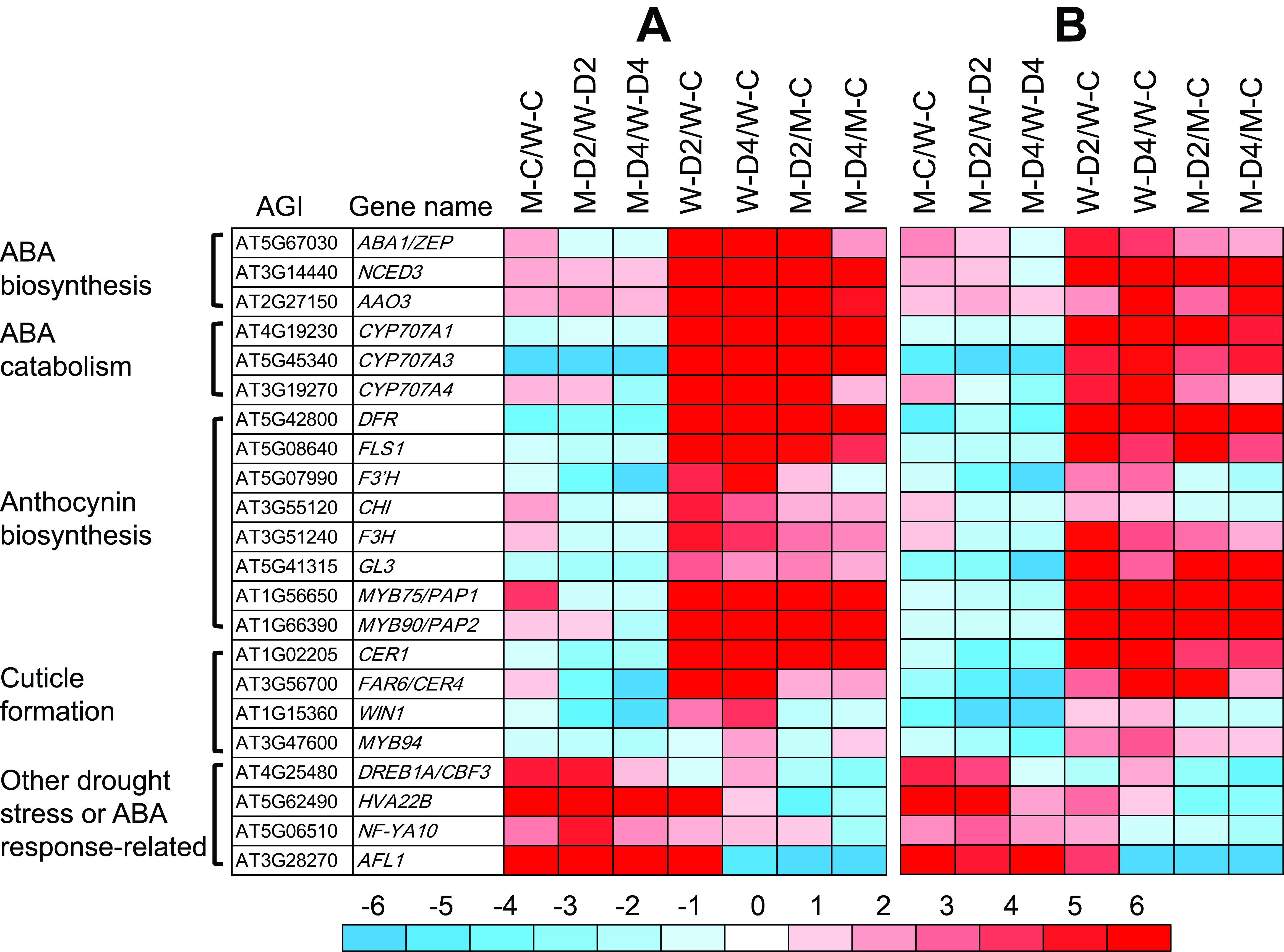

Supplement: S2 Fig — (A) Heatmap presentation indicates the fold-changes in expression of representative genes derived from microarray analysis. (B) Heatmap presentation indicates the fold-changes in expression of representative genes using qRT-PCR. Expression data were obtained from microarray analysis or qRT-PCR of 24-day-old Arabidopsis rosette leaf samples that were collected from 3 independent plants for microarray analysis (n = 3). UBQ10 was used as reference gene in qRT-PCR analysis. Relative expression levels are indicated by intensities of colors expressed in fold-change with saturation at 6. Red and blue colors indicate up- and downregulation, respectively. Note that not all data points shown in (a) passed the q-value < 0.05. M-C, kai2-2 well-watered control; M-D2, kai2-2 dehydrated 2 h; M-D4, kai2-2 dehydrated 4 h; W-C, WT well-watered control; W-D2, WT dehydrated 2 h; W-D4, WT dehydrated 4 h. (TIF) [file pgen.1007076.s002.tif]

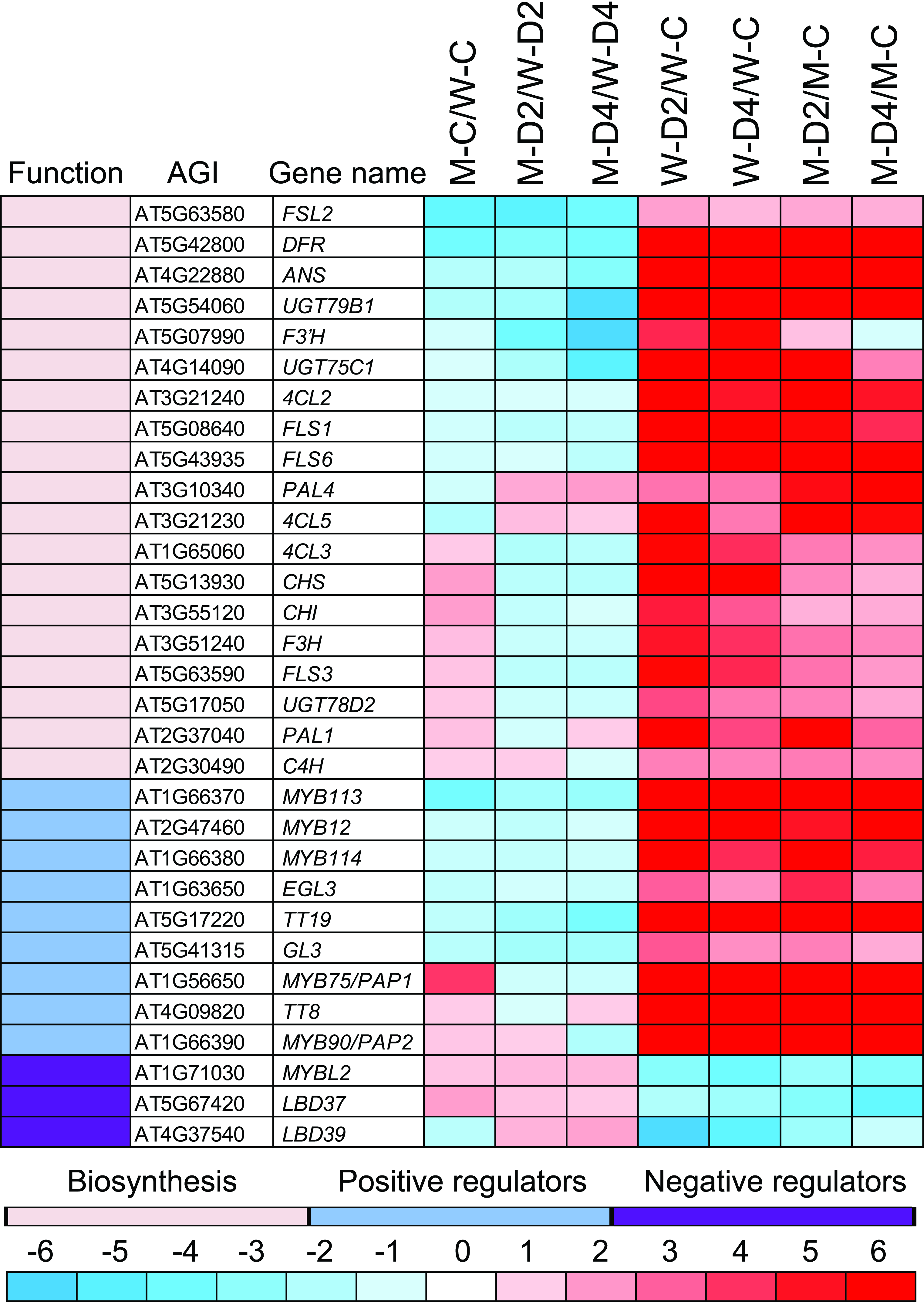

Supplement: S3 Fig — Heatmap presentation indicates the fold-changes in gene expression derived from microarray data. Relative expression levels are indicated by intensities of colors expressed in fold-change with saturation at 6. Red and blue colors indicate up- and downregulation, respectively. Note that not all data points shown passed the q-value < 0.05. M-C, kai2-2 well-watered control; M-D2, kai2-2 dehydrated 2 h; M-D4, kai2-2 dehydrated 4 h; W-C, WT well-watered control; W-D2, WT dehydrated 2 h; W-D4, WT dehydrated 4 h. (TIF) [file pgen.1007076.s003.tif]

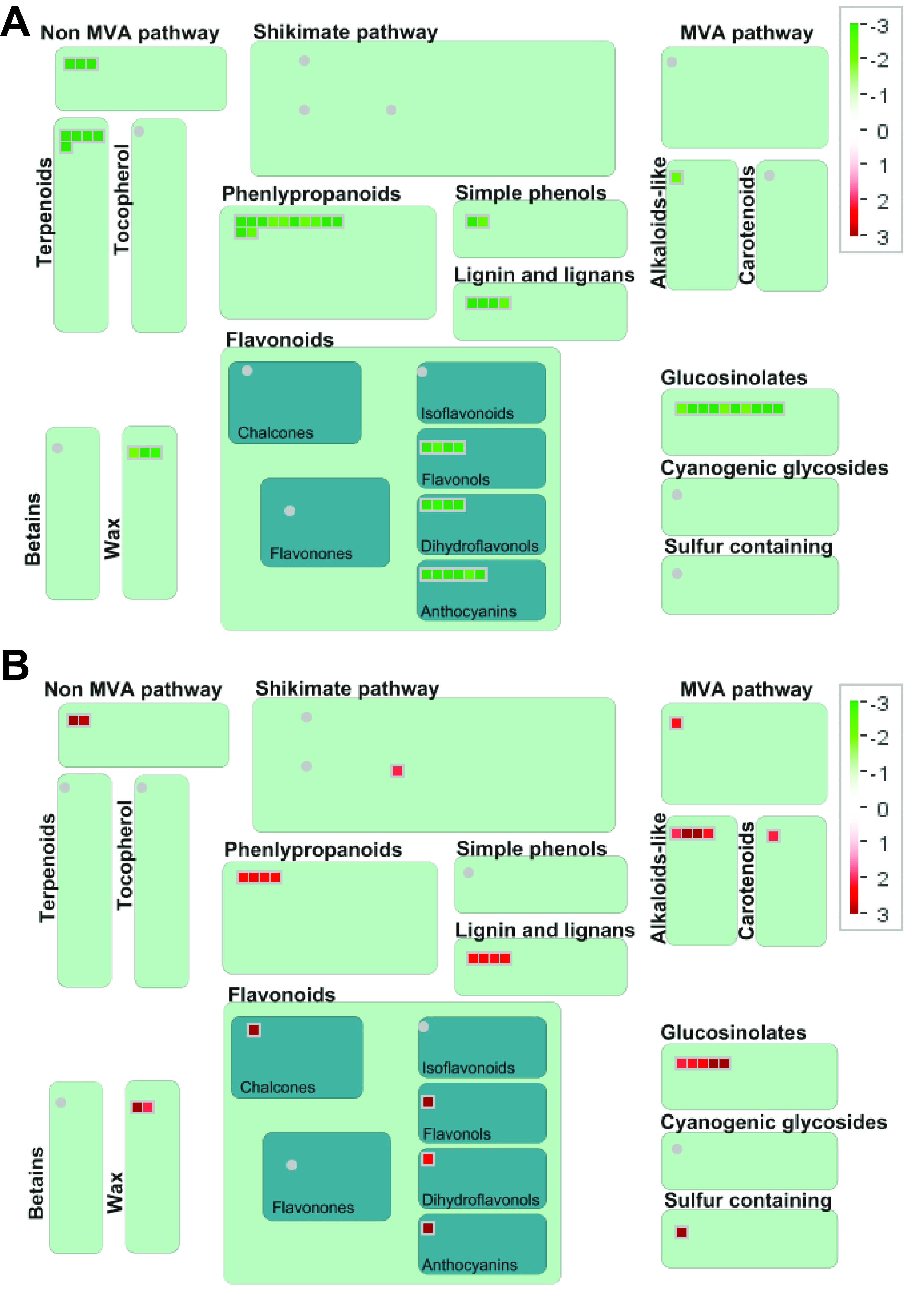

Supplement: S4 Fig — (A) Downregulated genes in M-C/W-C and/or M-D/W-D. (B) Upregulated genes in M-C/W-C and/or M-D/W-D. Green and red colors show down- and upregulation, respectively. Colored bars in each panel indicate fold-changes in gene expression. M-C/W-C, kai2-2 well-watered control versus WT well-watered control; M-D/W-D represents M-D2/W-D2 and/or M-D4/W-D4; M-C, kai2-2 well-watered control; M-D2, kai2-2 dehydrated 2 h; M-D4, kai2-2 dehydrated 4 h; W-C, WT well-watered control; W-D2, WT dehydrated 2 h; W-D4, WT dehydrated 4 h. For repetitive genes, their highest fold-change was used in the analysis. (TIF) [file pgen.1007076.s004.tif]

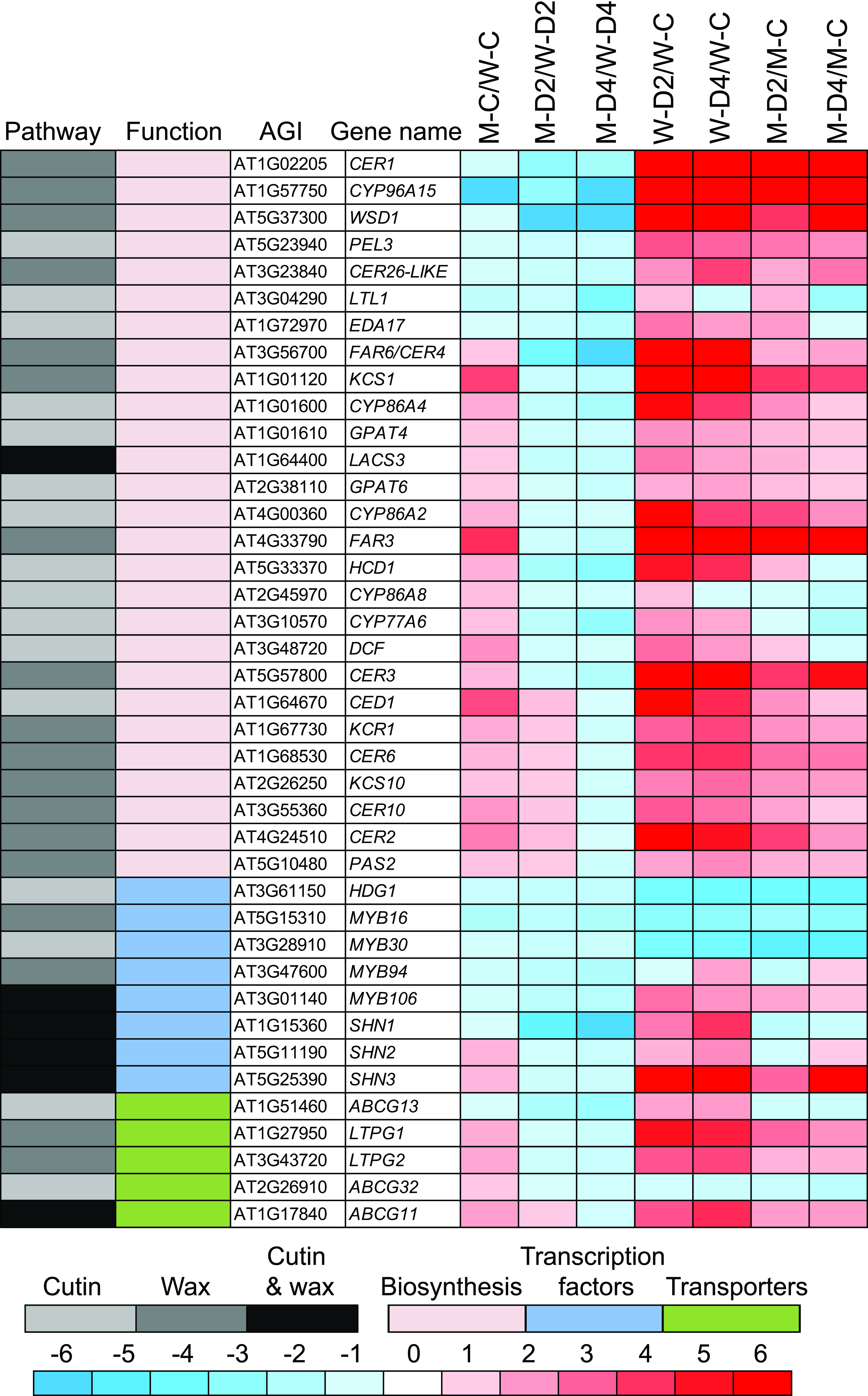

Supplement: S5 Fig — Heatmap presentation indicates the fold-changes in gene expression derived from microarray data. Relative expression levels are indicated by intensities of colors expressed in fold-change with saturation at 6. Red and blue colors indicate up- and downregulation, respectively. Note that not all data points shown passed the q-value < 0.05. M-C, kai2-2 well-watered control; M-D2, kai2-2 dehydrated 2 h; M-D4, kai2-2 dehydrated 4 h; W-C, WT well-watered control; W-D2, WT dehydrated 2 h; W-D4, WT dehydrated 4 h. (TIF) [file pgen.1007076.s005.tif]

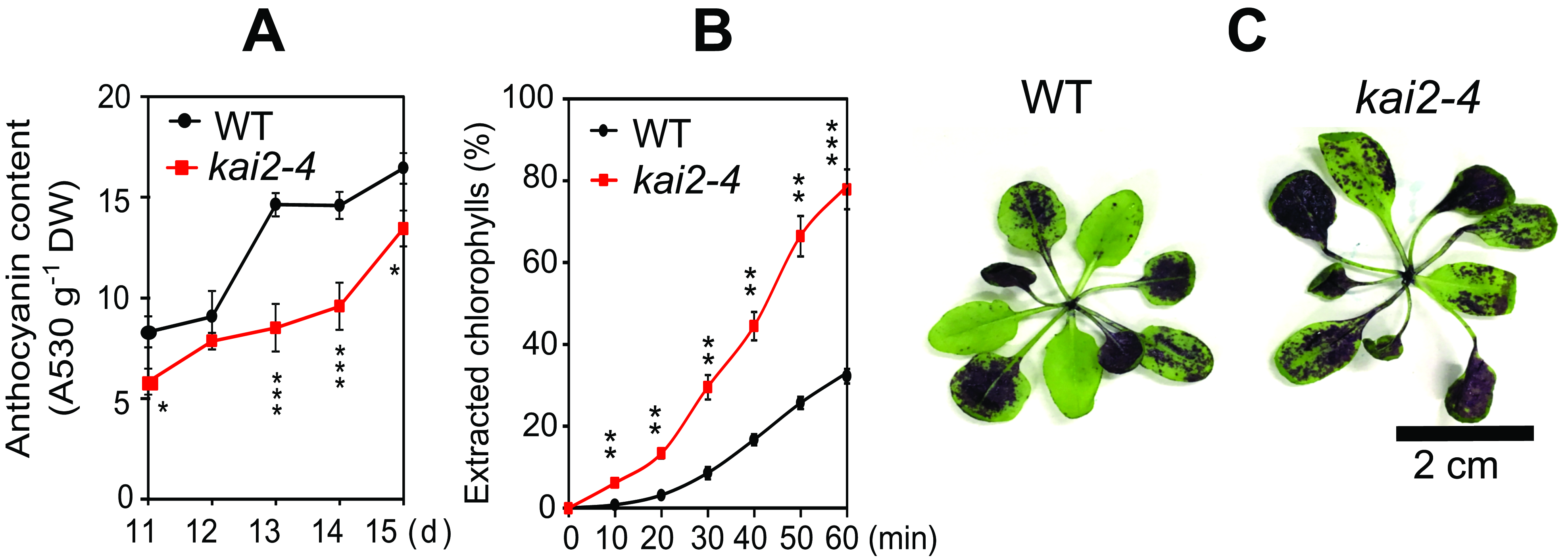

Supplement: S6 Fig — (A) Anthocyanin content in kai2-4 and WT plants under drought conditions. Data represent the means and standard errors (n = 4 plants). (B) Chlorophyll leaching from rosette leaves of 28-day-old kai2-4 and WT plants at different time periods. Data represent the means and standard errors (n = 3 plants/genotype). (C) Toluidine blue staining patterns of rosette leaves of 28-day-old kai2-4 and WT plants. Asterisks indicate significant differences as determined by a Student’s t-test, *P < 0.05; **P < 0.01; ***P < 0.001. (TIF) [file pgen.1007076.s006.tif]

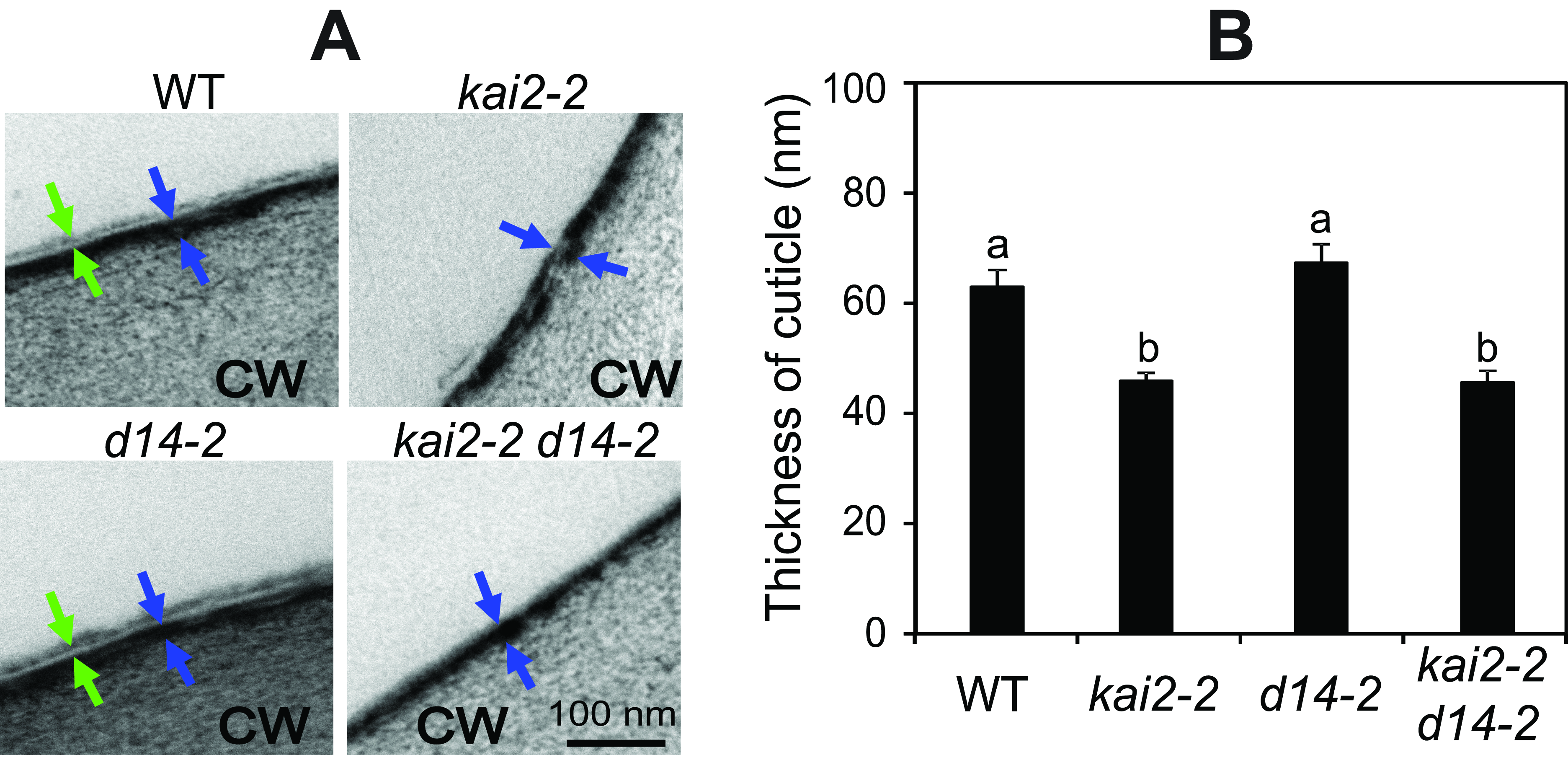

Supplement: S7 Fig — (A) Thickness of cuticle of the fifth leaves (adaxial side) derived from kai2-2, d14-2, kai2-2 d14-2 and WT plants. Blue arrows indicate cuticular layer (electron-dense, darker-staining layer) and green arrows indicate wax-rich cuticle proper (electron-translucent layer). (B) Transmission electron microscope images of the surface of the fifth leaves (adaxial side) derived from kai2-2, d14-2, kai2-2 d14-2 and WT plants. CW, cell wall. Data represent the means and standard errors (n = 3 biological replicates). Different letters above the error bars indicate significant differences (P < 0.05) among the genotypes according to a Tukey's honest significant difference test. (TIF) [file pgen.1007076.s007.tif]
